# Two Randomized Trials of Low-Dose Calcium Supplementation in Pregnancy

**DOI:** 10.1056/NEJMoa2307212

**Published:** 2024-01-11

**Authors:** Pratibha Dwarkanath, Alfa Muhihi, Christopher R. Sudfeld, Blair J. Wylie, Molin Wang, Nandita Perumal, Tinku Thomas, Shabani M. Kinyogoli, Mohamed Bakari, Ryan Fernandez, John-Michael Raj, Ndeniria O. Swai, Nirmala Buggi, Rani Shobha, Mary M. Sando, Christopher P. Duggan, Honorati M. Masanja, Anura V. Kurpad, Andrea B. Pembe, Wafaie W. Fawzi

**Affiliations:** St. John’s Research Institute, Bangalore, India; Africa Academy for Public Health, Dar es Salaam, Tanzania; Harvard T.H. Chan School of Public Health, Boston; Columbia University Medical Center, New York; Harvard T.H. Chan School of Public Health, Boston, Harvard Medical School, Boston; Harvard T.H. Chan School of Public Health, Boston, University of South Carolina, Columbia; St. John’s Medical College, Bangalore, India; Africa Academy for Public Health, Dar es Salaam, Tanzania; Muhimbili University of Health and Allied Sciences, Dar es Salaam, Tanzania; St. John’s Research Institute, Bangalore, India; St. John’s Medical College, Bangalore, India; Dar es Salaam Regional Medical Office of Health, Dar es Salaam, Tanzania; Bruhat Bengaluru Mahanagara Palike, Bangalore, India; Bruhat Bengaluru Mahanagara Palike, Bangalore, India; Africa Academy for Public Health, Dar es Salaam, Tanzania; Harvard T.H. Chan School of Public Health, Boston, Harvard Medical School, Boston, Boston Children’s Hospital, Boston; Ifakara Health Institute, Dar es Salaam, Tanzania; St. John’s Medical College, Bangalore, India; Muhimbili University of Health and Allied Sciences, Dar es Salaam, Tanzania; Harvard T.H. Chan School of Public Health, Boston

## Abstract

**BACKGROUND:**

The World Health Organization recommends 1500 to 2000 mg of calcium daily as supplementation, divided into three doses, for pregnant persons in populations with low dietary calcium intake in order to reduce the risk of preeclampsia. The complexity of the dosing scheme, however, has led to implementation barriers.

**METHODS:**

We conducted two independent randomized trials of calcium supplementation, in India and Tanzania, to assess the noninferiority of a 500-mg daily dose to a 1500-mg daily dose of calcium supplementation. In each trial, the two primary outcomes were preeclampsia and preterm birth, and the noninferiority margins for the relative risks were 1.54 and 1.16, respectively.

**RESULTS:**

A total of 11,000 nulliparous pregnant women were included in each trial. The cumulative incidence of preeclampsia was 3.0% in the 500-mg group and 3.6% in the 1500-mg group in the India trial (relative risk, 0.84; 95% confidence interval [CI], 0.68 to 1.03) and 3.0% and 2.7%, respectively, in the Tanzania trial (relative risk, 1.10; 95% CI, 0.88 to 1.36) — findings consistent with the noninferiority of the lower dose in both trials. The percentage of live births that were preterm was 11.4% in the 500-mg group and 12.8% in the 1500-mg group in the India trial (relative risk, 0.89; 95% CI, 0.80 to 0.98), which was within the noninferiority margin of 1.16; in the Tanzania trial, the respective percentages were 10.4% and 9.7% (relative risk, 1.07; 95% CI, 0.95 to 1.21), which exceeded the noninferiority margin.

**CONCLUSIONS:**

In these two trials, low-dose calcium supplementation was noninferior to high-dose calcium supplementation with respect to the risk of preeclampsia. It was noninferior with respect to the risk of preterm live birth in the trial in India but not in the trial in Tanzania. (Funded by the Bill and Melinda Gates Foundation and others; ClinicalTrials.gov number, NCT03350516; Clinical Trials Registry–India number, CTRI/2018/02/012119; and Tanzania Medicines and Medical Devices Authority Trials Registry number, TFDA0018/CTR/0010/5).

HYPERTENSIVE DISORDERS OF PREGnancy, which include preeclampsia, complicate 2 to 8% of pregnancies and are estimated to cause 45,000 maternal deaths annually.^1,2^ These disorders are also associated with an increased risk of preterm birth, the leading cause of death among children worldwide.^3,4^ Therefore, the implementation of effective strategies to prevent hypertensive disorders of pregnancy and preterm birth will be essential for countries to reach the maternal and child mortality targets of the United Nations Sustainable Development Goals by 2030.

Calcium supplementation in pregnancy has been recommended by the World Health Organization (WHO) since 2011 to reduce the risk of preeclampsia in populations with low dietary calcium intake.^5,6^ In placebo-controlled trials, high-dose calcium supplementation of at least 1000 mg per day reduced the risk of preeclampsia by more than half and the risk of preterm birth by 24%; the reduction in the risk of preeclampsia appeared to be greater in trials that had been conducted in populations with low-calcium diets.^7^ On the basis of this evidence, the WHO has recommended calcium supplementation of 1500 to 2000 mg per day, divided into three doses, taken a few hours apart from iron–folic acid supplements.^6^ In more than a decade since the 2011 recommendation, only a few countries have implemented routine high-dose calcium supplementation in pregnancy, mainly owing to adherence concerns and high programmatic costs associated with the complex dosing scheme.^8,9^ Trials of low-dose calcium supplementation of less than 1000 mg per day in pregnancy, most of which evaluated a single 500-mg calcium supplement per day as compared with placebo and have had relatively small sample sizes, have generally shown a magnitude of reduction in the risks of preeclampsia and preterm birth similar to that seen in the trials of high-dose supplementation.^7^

We hypothesized that low-dose calcium supplementation in pregnancy may be as efficacious as high-dose supplementation with respect to the incidence of preeclampsia and preterm birth. We conducted two randomized, noninferiority trials to compare the efficacy of 500 mg of calcium supplementation per day with 1500 mg per day in India and Tanzania.

## METHODS

### TRIAL DESIGNS

We conducted two independent, individually randomized, parallel-group, double-blind, noninferiority trials of low-dose calcium supplementation as compared with high-dose calcium supplementation in nulliparous pregnant women in India and Tanzania. The trials were designed to have similar interventions, methods, and outcome definitions but were independently powered and were planned to be analyzed separately. The methods for the trials have been published previously.^10^ The third, fifth, and last authors vouch for the accuracy and completeness of the data and for fidelity of the trial to the protocol, which is available with the full text of this article at NEJM.org.

Participants were enrolled at health clinics in Bangalore, India, and in Dar es Salaam, Tanzania. Participants were adult (≥18 years of age) nulliparous pregnant women who were at less than 20 weeks’ gestation (according to the date of the last menstrual period), who intended to stay in the trial area until 6 weeks post partum, and who provided written informed consent. Women were excluded from enrollment if they had a history, signs, or symptoms of nephrolithiasis; had a history of parathyroid disorder or had undergone thyroidectomy; or had a disease for which digoxin, phenytoin, or tetracycline therapy was indicated.

### INTERVENTIONS

Participants in India and Tanzania were randomly assigned to receive either 500 mg or 1500 mg of elemental calcium supplementation to be taken orally each day until delivery. The 500-mg calcium supplementation group received one tablet that contained 500 mg of elemental calcium as calcium carbonate and two placebo tablets each day, and the 1500-mg calcium supplementation group received three 500-mg tablets each day. In India, vitamin D_3_ is recommended to be taken with calcium supplements, and therefore the two groups in the India trial also received 250 IU of vitamin D_3_ per day.^11^ There was no vitamin D_3_ added to the tablets in the Tanzania trial.

In each trial, pregnant participants received a 35-day supply of tablets in blister packs at each trial visit. The blister packs contained a 7-day supply of the trial tablets, with columns indicating the tablet to be taken in the morning, midday, and evening. Blister packs were delivered to the homes of pregnant participants who could not attend follow-up visits at trial clinics. Adherence was assessed by means of pill counts. Influx Healthcare (in Maharashtra, India) manufactured the tablets for both trials. The company was paid full price for the tablets and had no role in the trial design, data collection, the interpretation of the results, or the writing of the manuscript.

### RANDOMIZATION AND BLINDING

Randomization procedures were independently conducted in the India and Tanzania trials. The assignment sequence for each trial was generated by a statistician not otherwise involved in the trial by means of a computer-generated list of participant identification numbers with block randomization, stratified according to trial clinic. At the randomization visit, participants were assigned the next available participant identification number, which corresponded to a set of prelabeled blister packs. All the calcium and placebo tablets were identical in appearance, taste, and smell and were packaged in indistinguishable blister packs. The codes linking participant identification numbers with the randomly assigned calcium supplementation groups were broken after the blinded analyses were conducted.

### DATA COLLECTION AND OUTCOMES

Participants had follow-up clinic visits each month during pregnancy, at delivery, and at 6 weeks post partum. Pregnant participants’ baseline dietary intake was assessed by means of an open-ended 24-hour diet recall. Pregnant participants had a finger-prick blood sample obtained at the time of randomization and at 32 weeks’ gestation to assess hemoglobin concentrations.

The primary efficacy outcomes were preeclampsia and preterm birth. Preeclampsia was defined as the meeting of at least one of the following criteria from 20 weeks’ gestation to delivery: gestational hypertension and proteinuria among participants without chronic hypertension, gestational proteinuria among participants with chronic hypertension (superimposed preeclampsia), clinical diagnosis of preeclampsia, or the development of preeclampsia with severe features with or without proteinuria.^12,13^ Blood pressure was assessed at each trial visit by means of digital blood-pressure monitors. Dipsticks were used to assess the presence of protein in urine samples at each pregnancy visit and at delivery. Among patients without chronic hypertension, gestational hypertension was defined as a systolic blood pressure of at least 140mm Hg or a diastolic blood pressure of at least 90mm Hg as measured on two occasions at least 1hour apart or as severe hypertension with a systolic blood pressure of at least 160 mm Hg or a diastolic blood pressure of at least 110 mm Hg as measured on two occasions at least 1 minute apart in pregnancy or as measured on one occasion during the time of labor or delivery. Proteinuria was defined as a dipstick reading of at least 1+. Preeclampsia with severe features was defined as the presence of severe gestational hypertension (with or without proteinuria), eclampsia, end-organ dysfunction, clinical diagnosis of the HELLP (hemolysis, elevated liver-enzyme levels, and low platelet count) syndrome, the development of pulmonary edema, or new-onset central nervous system or visual symptoms.

Preterm birth was defined as a live birth before 37 weeks’ gestation as assessed by means of the best obstetrical estimate approach, which combined information from both the date of the last menstrual period and ultrasonographic assessment. The menstrual date–derived estimated date of delivery was changed to the ultrasound-derived estimated date of delivery if the dating differed by a prespecified number of days, which varied depending on the timing of ultrasonography. The methods for gestational-age dating in the trials have been described in greater detail previously.^10^

Secondary outcomes included gestational hypertension, preeclampsia with severe features, pregnancy-related death, fetal death, stillbirth (at ≥28 weeks’ gestation), low birth weight (<2500 g), small-for-gestational-age status at birth defined according to the INTERGROWTH-21st standard (<10th percentile regarding size for gestational age),^14^ and infant death before 42 days of age. Maternal hospitalization (excluding hospitalization for delivery) and third-trimester severe anemia (hemoglobin concentration, <7.0 g per deciliter) were evaluated as safety outcomes. Details of the outcome definitions are provided in Table S1 in the Supplementary Appendix, available at NEJM.org.

### STANDARD OF CARE AND ETHICS

All the participants in India and Tanzania received standard-care antenatal and postpartum services that were aligned with the country-specific antenatal care guidelines. In India, pregnant participants received daily supplements that contained 5 mg of folic acid during the first trimester and then supplements that contained 60 mg of elemental iron and 0.4 mg of folic acid during the second and third trimesters. In Tanzania, pregnant participants received daily iron–folic acid supplements that contained 60 mg of elemental iron and 0.4 mg of folic acid starting at the first antenatal care visit. The protocols of the trials were approved by institutional review boards. A data and safety monitoring board oversaw the trial.^10^

### STATISTICAL ANALYSIS

The India and Tanzania trials were independently powered and analyzed separately. Assuming a randomization ratio of 1:1, a one-sided test with a type I error of 0.05, and a 10% incidence of loss to follow-up or missing outcome data, we planned to enroll 11,000 pregnant participants in each trial. The cumulative incidences of preeclampsia and preterm birth were expected to be as low as 1.5% and 10%, respectively, in the high-dose supplementation group. The noninferiority margins for relative risk were 1.54 for preeclampsia and 1.16 for preterm birth (see the Methods section in the Supplementary Appendix).

The primary analyses used the intention-to-treat principle and included all the participants who had undergone randomization and had data available for the outcome of interest. Per-protocol analyses were also conducted for the primary outcomes (see below). All the models included fixed effects for trial clinic to account for the stratified randomization. Our protocol did not include a plan to adjust for the two primary efficacy outcomes in each trial, but we applied a Bonferroni correction to account for multiplicity (one-sided alpha of 0.025). Two-sided 95% confidence intervals are presented, which have an upper boundary equivalent to a one-sided 97.5% confidence interval.

Log-binomial models were used to estimate the relative risk of preeclampsia between the 500-mg group and the 1500-mg group. The per-protocol analyses of preeclampsia included pregnant participants who had more than 75% adherence to the assigned regimen and had a birth outcome assessed at 20 weeks’ gestation or later. Sensitivity analyses excluded participants with pregnancy loss and those who withdrew consent before 20 weeks’ gestation. Kaplan–Meier curves with gestational age as the time metric were also constructed.

The analyses of preterm birth were restricted to live births. Generalized estimating equations with log links and compound symmetry working correlation matrixes to account for multiple gestations were used to estimate the relative risks of preterm birth. Per-protocol analyses of preterm birth included live births among pregnant participants who had more than 75% adherence to the assigned regimen. Sensitivity analyses were restricted to singleton live births.

Log-binomial models were used to estimate relative risks for nonrepeatable secondary maternal outcomes, and generalized estimating equations were used for secondary infant outcomes in order to account for multiple gestations. Poisson regression models were used to estimate incidence rate ratios for the repeatable safety event of maternal hospitalization. Relative risks or incidence rate ratios with 95% confidence intervals are presented for all secondary and safety outcomes and were not adjusted for multiple comparisons. Fixed- and random-effects meta-analyses were conducted to produce pooled effect estimates. We conducted post hoc exploratory sensitivity analyses for early-onset preeclampsia (at <34 weeks’ gestation) and for preeclampsia onset at less than 37 weeks’ gestation, as well as an analysis of preterm birth that was restricted to participants with spontaneous birth. Statistical analyses were performed with the use of SAS software, version 9.4 (SAS Institute).

## RESULTS

### PARTICIPANTS

**Figure 1.**
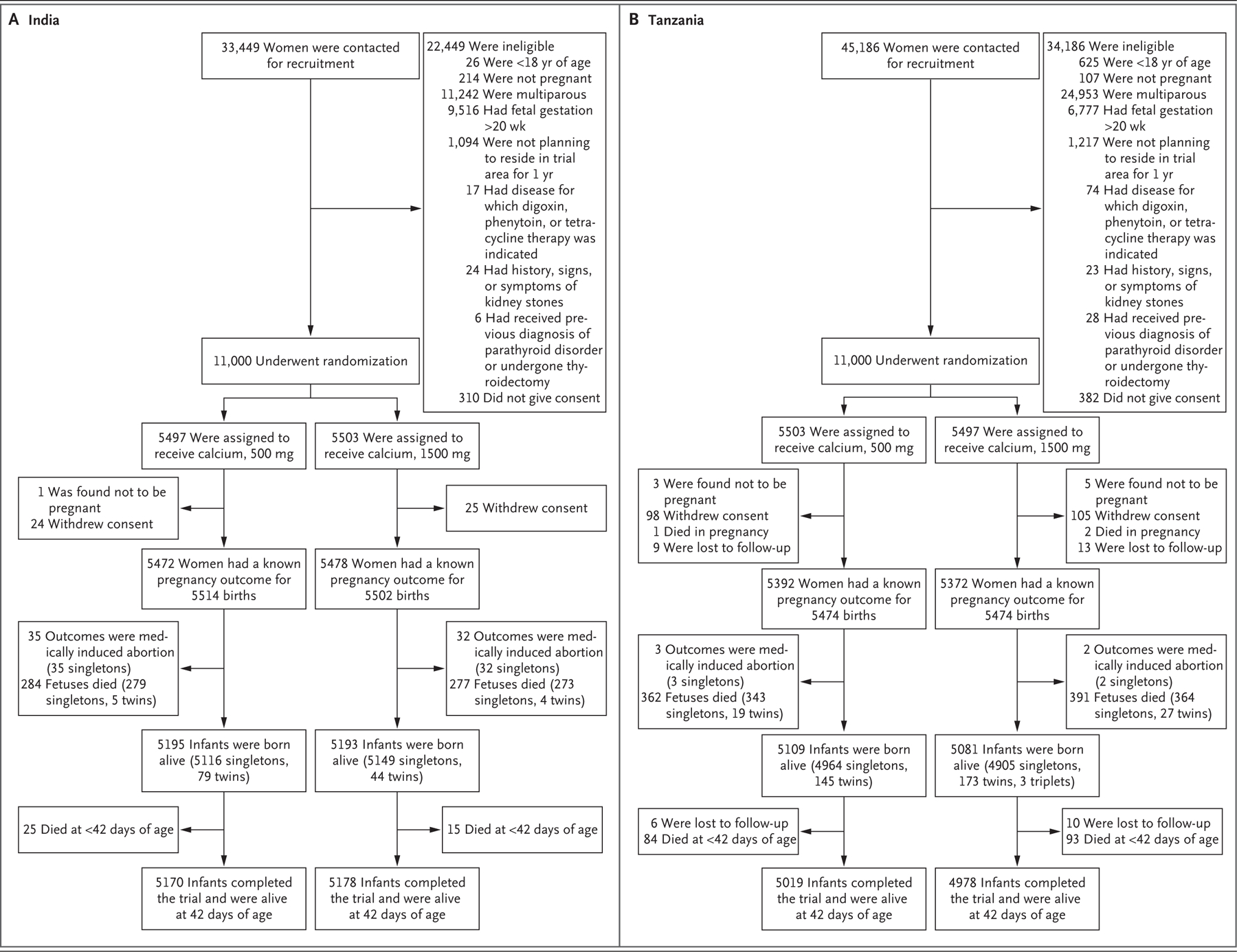
Follow-up of the Pregnant Women and Infants in the India and Tanzania Trials. In each trial, 11,000 nulliparous pregnant women were enrolled. The total number of births was greater than the number of women with pregnancy outcomes owing to multiple births (including twins and triplets).

In the India trial, from November 2018 through February 2022, we screened 33,449 women and enrolled 11,000 pregnant participants. In the Tanzania trial, from March 2019 through March 2022, we screened 45,186 women and enrolled 11,000 pregnant participants. The flow diagrams for the follow-up of the pregnant participants and infants in each trial are presented in Figure 1 and in Figures S1 and S2. Pregnancy outcomes were known for 99.5% of the pregnancies in the India trial and for 97.9% of those in the Tanzania trial. The median percentage adherence to calcium supplementation was 97.7% (interquartile range, 93.2 to 99.2) in the India trial and 92.3% (interquartile range, 82.7 to 97.1) in the Tanzania trial.

The characteristics of the participants at baseline were generally well-balanced between the groups in each trial (Table 1). In both trials, most of the pregnant participants were between 18 and 24 years of age and had normal blood pressure at baseline. The percentage of the participants with a baseline dietary calcium intake of less than 800 mg per day was approximately 87% in India and 67% in Tanzania.

### PRIMARY OUTCOMES

In the India trial, the cumulative incidence of preeclampsia was 3.0% in the 500-mg group and 3.6% in the 1500-mg group (relative risk, 0.84; 95% confidence interval [CI], 0.68 to 1.03); in the Tanzania trial, the cumulative incidence of preeclampsia was 3.0% in the 500-mg group and 2.7% in the 1500-mg group (relative risk, 1.10; 95% CI, 0.88 to 1.36) (Table 2). In both trials, the 500-mg dose of calcium was shown to be noninferior to the 1500-mg dose with regard to the risk of preeclampsia. Kaplan–Meier curves for the timing of preeclampsia are shown in Figures S3 and S4. In sensitivity analyses, there were no between-group differences in the incidence of early-onset preeclampsia at less than 34 weeks’ gestation or of preeclampsia onset at less than 37 weeks’ gestation (Table S2).

The incidence of preterm birth in the India trial was 11.4% in the 500-mg group and 12.8% in the 1500-mg group (relative risk, 0.89; 95% CI, 0.80 to 0.98); the incidence in the Tanzania trial was 10.4% in the 500-mg group and 9.7% in the 1500-mg group (relative risk, 1.07; 95% CI, 0.95 to 1.21). The findings were consistent with noninferiority in the India trial but not in the Tanzania trial.

Results of the per-protocol analyses, sensitivity analyses, and analyses with adjustment for potential baseline imbalance were similar to the primary analyses in each trial (Table 2 and Tables S3 and S4). There were no apparent between-group differences in the incidence of preterm birth in post hoc sensitivity analyses that were restricted to spontaneous births.

### SECONDARY AND SAFETY OUTCOMES

Results of the secondary and safety outcomes in the two trials are shown in Table 3. There was no evidence favoring the 1500-mg group over the 500-mg group with regard to the secondary or safety outcomes in either trial.

### META-ANALYSES

Fixed- and random-effects meta-analyses of the outcomes in the two trials did not indicate a difference between the 500-mg group and 1500-mg group with regard to the risks of preeclampsia, preterm birth, and the secondary and safety outcomes. Details are provided in Figures S5 through S15.

## DISCUSSION

In two large, randomized trials conducted in India and Tanzania, each of which enrolled 11,000 nulliparous pregnant participants, the use of low-dose calcium supplementation at a dose of 500 mg per day was noninferior to standard high-dose supplementation of 1500 mg per day with respect to the incidence of preeclampsia. For preterm birth, the use of low-dose calcium supplementation was noninferior in the India trial but did not show noninferiority in the Tanzania trial. Meta-analyses of data from the two trials were consistent, with no material difference between low-dose and high-dose supplementation for the primary, secondary, and safety outcomes.

Calcium supplementation may lower blood pressure by reducing parathyroid hormone release and intracellular calcium, resulting in reduced vascular smooth-muscle contractility.^15^ By means of a similar mechanism, calcium supplementation could also reduce uterine smooth-muscle contractility and prevent preterm labor.^16,17^ Placebo-controlled trials that evaluated high-dose calcium supplementation regimens at doses of 1500 to 2000 mg per day informed the WHO guidelines.^7^ Our two trials showed that low-dose supplementation with 500 mg of calcium per day was noninferior to high-dose supplementation for the prevention of preeclampsia. A review of diet studies suggests that pregnant populations in low- and middle-income countries have a mean calcium intake of approximately 600 mg per day.^18^ In these contexts, and in our trial populations, which had a median intake of approximately 400 mg per day, an additional 500-mg calcium supplement would fill the nutrient gap for most pregnant persons.^19^ There is also limited evidence that calcium supplementation before conception and in early pregnancy may provide greater beneficial effects on preeclampsia than supplementation initiated after 20 weeks’ gestation.^20^ In our trials, only approximately one third of the participants started calcium supplementation in the first trimester of pregnancy. Furthermore, the benefit of coadministration of calcium supplements with vitamin D, aspirin, or other cointerventions for the prevention of preeclampsia remains unclear.^21^

We found that low-dose calcium supplementation was noninferior to high-dose supplementation for preterm birth in the India trial; however, this was not the case in the Tanzania trial, in which the upper boundary of the confidence interval crossed the noninferiority margin. In the Tanzania trial, the risk of preterm birth in the 1500-mg group was slightly less than predicted in the power calculations, and therefore the confidence intervals were somewhat wider than expected.

The high-dose calcium supplementation regimen that is currently recommended by the WHO requires pregnant populations with low dietary calcium intake to take four nutritional supplements per day (calcium three times daily plus a daily iron–folic acid or multivitamin supplement); adherence to taking a drug or supplement decreases as the number of doses per day increases.^22^ Fewer supplements per day may also make it easier to take iron–folic acid and calcium tablets at different times. Furthermore, the cost of a three-tablet calcium supplementation regimen per pregnancy is estimated to be $11.50, which far exceeds the approximate $1 cost per pregnancy for iron–folic acid supplementation.^6^ The 500-mg dose that we studied as a comparator reduces the pill burden and would be expected to reduce program costs.

Our trials have some limitations. The two trials used the best obstetrical estimate for gestational age on the basis of the reported last menstrual period and fetal ultrasonography; however, we cannot rule out some measurement error and misclassification for preterm birth. We also assessed participant dietary intake with the 24-hour diet recall method, which is prone to measurement error owing to day-to-day variation in diets.^23^ However, the dietary data support the assumption that the trials were conducted in populations with low dietary calcium intake. Given the noninferiority focus of the trials and ethics considerations, we did not include a placebo group and cannot compare outcome risks with regard to no calcium supplementation. Our trials also enrolled only nulliparous pregnant women owing to their increased risk of preeclampsia.^24^ As a result, the trial populations generally included young participants who had a low risk of chronic hypertension. Therefore, care should be taken when considering the generalizability of our findings to other pregnant populations. The representativeness of the trial participants is shown in Table S5.

Overall, our findings in these two trials showed that low-dose calcium supplementation in pregnancy was noninferior to high-dose supplementation with respect to the risk of preeclampsia. The trial in India, but not the one in Tanzania, showed that low-dose supplementation was noninferior to high-dose supplementation with respect to the risk of preterm birth.

## Supplementary Material

supplement

## Figures and Tables

**Table 1.** Characteristics of the Participants at Baseline in the India and Tanzania Trial Populations.*

Characteristic	India Trial (N = 11,000)		Tanzania Trial (N = 11,000)		
	500 mg Calcium (N = 5497)	1500 mg Calcium (N = 5503)	500 mg Calcium (N = 5503)		1500 mg Calcium (N = 5497)
Maternal age — no. (%)					
18–24 yr	3372 (61.3)	3436 (62.4)	3882 (70.5)		3879 (70.6)
25–29 yr	1616 (29.4)	1550 (28.2)	1299 (23.6)		1257 (22.9)
≥30 yr	509 (9.3)	517 (9.4)	322 (5.9)		361 (6.6)
Maternal education — no./total no. (%)					
No formal education	162/5496 (2.9)	168/5502 (3.1)	82/5496 (1.5)		94/5491 (1.7)
Primary education	215/5496 (3.9)	199/5502 (3.6)	1802/5496 (32.8)		1744/5491 (31.8)
Secondary or higher education	5119/5496 (93.1)	5135/5502 (93.3)	3612/5496 (65.7)		3653/5491 (66.5)
Wealth quintile — no./total no. (%)					
Quintile 1: Poorest	1104/5496 (20.1)	1096/5502 (19.9)	1099/5503 (20.0)		1099/5497 (20.0)
Quintile 2: Second poorest	1076/5496 (19.6)	1130/5502 (20.5)	1109/5503 (20.2)		1093/5497 (19.9)
Quintile 3: Middle	1122/5496 (20.4)	1071/5502 (19.5)	1119/5503 (20.3)		1081/5497 (19.7)
Quintile 4: Second richest	1077/5496 (19.6)	1131/5502 (20.6)	1055/5503 (19.2)		1145/5497 (20.8)
Quintile 5: Richest	1117/5496 (20.3)	1074/5502 (19.5)	1121/5503 (20.4)		1079/5497 (19.6)
Gestational age — no. (%)†					
<13 wk 0 days	1675 (30.5)	1718 (31.2)	1598 (29.0)		1699 (30.9)
13 wk 0 days to 16 wk 6 days	2231 (40.6)	2170 (39.4)	2085 (37.9)		2070 (37.7)
17 wk 0 days to 20 wk 0 days	1591 (28.9)	1615 (29.3)	1820 (33.1)		1728 (31.4)
Maternal height <155.0 cm — no./total no. (%)	3241/5496 (59.0)	3295/5502 (59.9)	1853/5503 (33.7)		1824/5497 (33.2)
Body-mass index — no./total no. (%)‡					
<18.5	898/5496 (16.3)	886/5502 (16.1)	452/5503 (8.2)		440/5497 (8.0)
18.5–24.9	2846/5496 (51.8)	2869/5502 (52.1)	3202/5503 (58.2)		3162/5497 (57.5)
25.0–29.9	1238/5496 (22.5)	1225/5502 (22.3)	1269/5503 (23.1)		1306/5497 (23.8)
≥30.0	514/5496 (9.4)	522/5502 (9.5)	578/5503 (10.5)		588/5497 (10.7)
Hemoglobin concentration — no. (%)					
≥11.0 g/dl	3018 (54.9)	3034 (55.1)	3241 (58.9)		3233 (58.8)
10.0–10.9 g/dl	1762 (32.1)	1679 (30.5)	1534 (27.9)		1531 (27.9)
7.0–9.9 g/dl	687 (12.5)	766 (13.9)	728 (13.2)		732 (13.3)
<7.0 g/dl	15 (0.3)	13 (0.2)	0		1 (<0.1)
Living with HIV infection — no. (%)	NA	NA	107 (1.9)		89 (1.6)
Family history of hypertension — no./total no. (%)	326/5496 (5.9)	325/5502 (5.9)	1025/5503 (18.6)		1050/5497 (19.1)
High blood pressure — no. (%)§	34/5482 (0.6)	45/5492 (0.8)	14/5503 (0.3)		18/5497 (0.3)
Taking antihypertensive drug — no. (%)	1/5496 (<0.1)	3/5502 (0.1)	10/5503 (0.2)		9/5497 (0.2)
Median caloric intake (IQR) — kcal¶	1169 (946–1421)	1166 (956–1421)	2862 (2144–3600)		2882 (2206–3647)
Median dietary calcium intake (IQR) — mg/day¶	431 (292–629)	440 (297–633)	413 (193–1143)		413 (190–1233)
Dietary calcium intake <800 mg/day — no./total no. (%)‖	4774/5497 (86.8)	4802/5503 (87.3)	3046/4535 (67.2)		3035/4526 (67.1)

* HIV denotes human immunodeficiency virus, IQR interquartile range, and NA not available.

† Gestational age was defined on the basis of the reported date of the last menstrual period (enrollment criterion). In the India trial, 11 participants (6 in the 500-mg group and 5 in the 1500-mg group) underwent randomization between 20 weeks 1 day of gestation age and 20 weeks 6 days of gestational age.

‡ The body-mass index is the weight in kilograms divided by the square of the height in meters.

§ High blood pressure was defined as a systolic blood pressure of at least 140 mm Hg or a diastolic blood pressure of at least 90 mm Hg.

¶ In the Tanzania trial, data on dietary intake were missing for 968 participants in the 500-mg group and for 971 in the 1500-mg group.

‖ An intake of 800 mg of calcium per day is the U.S. Institute of Medicine estimated average requirement for calcium intake among pregnant or lactating persons 19 to 50 years of age.

**Table 2.** Primary Efficacy Outcomes in the India and Tanzania Trials.*

Outcome	India Trial				Tanzania Trial			
	500 mg Calcium	1500 mg Calcium	Relative Risk (95% CI)	P Value for Noninferiority	500 mg Calcium	1500 mg Calcium	Relative Risk (95% CI)	P Value for Noninferiority
**Preeclampsia**								
Primary intention-to-treat analysis	164/5497 (3.0)	196/5503 (3.6)	0.84 (0.68–1.03)	<0.001	165/5503 (3.0)	150/5497 (2.7)	1.10 (0.88–1.36)	<0.001
Per-protocol analysis†	156/5027 (3.1)	184/5022 (3.7)	0.85 (0.69–1.05)		148/4420 (3.3)	128/4448 (2.9)	1.16 (0.92–1.46)	
Sensitivity analysis excluding participants who had pregnancy loss or withdrew before 20 wk of gestation	164/5397 (3.0)	196/5408 (3.6)	0.84 (0.69–1.03)		165/5361 (3.1)	150/5297 (2.8)	1.09 (0.87–1.35)	
**Preterm birth** ‡								
Primary intention-to-treat analysis	593/5195 (11.4)	665/5193 (12.8)	0.89 (0.80–0.98)	<0.001	531/5109 (10.4)	493/5081 (9.7)	1.07 (0.95–1.21)	0.10
Per-protocol analysis§	552/4852 (11.4)	629/4842 (13.0)	0.87 (0.78–0.97)		448/4279 (10.5)	430/4323 (9.9)	1.06 (0.93–1.20)	
Sensitivity analysis involving single-ton live births	559/5116 (10.9)	637/5149 (12.4)	0.88 (0.79–0.98)		448/4964 (9.0)	412/4905 (8.4)	1.07 (0.95–1.22)	

* P values are for noninferiority. A post hoc Bonferroni correction was applied to the primary efficacy outcomes within each trial to account for tests of the two efficacy outcomes; two-sided 95% confidence intervals are shown, and a P value for noninferiority of less than 0.025 was considered to indicate statistical significance.

† The per-protocol analysis for preeclampsia included all the pregnant participants who had more than 75% adherence to the randomly assigned regimen, had a pregnancy of at least 20 weeks’ gestation, and had a delivery outcome assessed (excluding withdrawal and loss to follow-up in pregnancy).

‡ Gestational age was determined on the basis of the best obstetrical estimate.

§ The per-protocol analysis for preterm birth included live births born to pregnant participants who had more than 75% adherence to the randomly assigned regimen.

**Table 3.** Secondary and Safety Outcomes in the India and Tanzania Trials.*

Outcome	India Trial			Tanzania Trial		
	500 mg Calcium	1500 mg Calcium	Relative Risk or Incidence Rate Ratio (95% CI)	500 mg Calcium	1500 mg Calcium	Relative Risk or Incidence Rate Ratio (95% CI)
**Secondary outcomes**						
Gestational hypertension — no./total no. (%)†	176/5477 (3.2)	207/5468 (3.8)	0.85 (0.70–1.03)	225/5481 (4.1)	220/5469 (4.0)	1.02 (0.85–1.22)
Preeclampsia with severe features — no./total no. (%)	61/5497 (1.1)	97/5503 (1.8)	0.63 (0.46–0.87)	100/5503 (1.8)	94/5497 (1.7)	1.06 (0.80–1.40)
Pregnancy-related death — no./total no. (%)	2/5497 (0.04)	2/5503 (0.04)	1.00 (0.14–7.10)	4/5503 (0.1)	3/5497 (0.1)	1.33 (0.30–5.95)
Fetal death — no./total no. (%)	284/5479 (5.2)	277/5470 (5.1)	1.03 (0.87–1.20)	362/5471 (6.6)	391/5472 (7.2)	0.93 (0.81–1.07)
Stillbirth at ≥28 wk of gestation — no./total no. (%)	110/5305 (2.1)	120/5313 (2.3)	0.92 (0.71–1.18)	165/5274 (3.1)	158/5239 (3.0)	1.05 (0.85–1.30)
Birth weight <2500 g — no./total no. (%)	898/5195 (17.3)	910/5193 (17.5)	0.98 (0.90–1.06)	448/5095 (8.8)	438/5066 (8.7)	1.03 (0.90–1.18)
Small-for-gestational-age status <10th percentile — no./total no. (%)	1703/5195 (32.8)	1777/5193 (34.2)	0.96 (0.90–1.01)	1133/5095 (22.2)	1112/5066 (22.0)	1.02 (0.94–1.09)
Infant death at <42 days — no./total no. (%)	25/5195 (0.5)	15/5193 (0.3)	1.60 (0.84–3.06)	84/5109 (1.6)	93/5081 (1.8)	0.90 (0.67–1.22)
**Safety outcomes**						
Maternal hospitalization — no. of hospitalizations/no. of person-mo	11/43,223	24/43,332	0.46 (0.22–0.94)	40/38,164	25/38,705	1.60 (0.97–2.63)
Maternal third-trimester severe anemia — no./total no. (%)‡	1/4475 (<0.1)	2/4478 (<0.1)	0.50 (0.05–5.52)	0/4229	0/4215	—

* Relative risks are shown for all secondary and safety outcomes except for the repeatable safety event of maternal hospitalization, for which incidence rate ratios are shown. For these nonprimary outcome analyses, the 95% confidence intervals are not adjusted for multiplicity and should not be used to infer definitive treatment effects.

† The analysis of gestational hypertension excluded participants with chronic hypertension (20 participants in the 500-mg group and 35 in the 1500-mg group in the India trial, and 22 and 28 participants, respectively, in the Tanzania trial).

‡ Severe anemia was defined as a hemoglobin concentration of less than 7.0 g per deciliter.
