# Supplementary material for: Two Randomized Trials of Low-Dose Calcium Supplementation in Pregnancy
Source: N Engl J Med. Author manuscript; Available in PMC 2024 Mar 8. (PMC10921922; doi:10.1056/NEJMoa2307212)
Supplement: supplement [file NIHMS1960298-supplement-supplement.pdf]

Supplementary Appendix

Supplement to: Dwarkanath P, Muhihi A, Sudfeld CR, et al. Two randomized trials of low-dose calcium supplementation in pregnancy. N Engl J Med 2024;390:143-53. DOI: 10.1056/NEJMoa2307212

This appendix has been provided by the authors to give readers additional information about the work.

Supplementary Appendix

List of Authors 2

Methods 3

Figure S1. India trial: Flow diagram for pregnant participants 4

Figure S2. Tanzania trial: Flow diagram for pregnant participants 5

Figure S3. India trial: Kaplan-Meir Curve of the cumulative incidence of preeclampsia by gestational age in weeks stratified by randomized group. 6

Figure S4. Tanzania trial: Kaplan-Meir Curve of the cumulative incidence of preeclampsia by gestational age in weeks stratified by randomized group. 7

Figure S5. Fixed and random effects meta-analysis of India and Tanzania trials: Preeclampsia. 8

Figure S6. Fixed and random effects meta-analysis of India and Tanzania trials: Preterm birth. 9

Figure S7. Fixed and random effects meta-analysis of India and Tanzania trials: Gestational hypertension. 10

Figure S8. Fixed and random effects meta-analysis of India and Tanzania trials: Preeclampsia with severe features. 11

Figure S9. Fixed and random effects meta-analysis of India and Tanzania trials: Maternal hospitalization. 12

Figure S10. Fixed and random effects meta-analysis of India and Tanzania trials: Maternal death. 13

Figure S11. Fixed and random effects meta-analysis of India and Tanzania trials: Fetal death. 14

Figure S12. Fixed and random effects meta-analysis of India and Tanzania trials: Stillbirth. 15

Figure S13. Fixed and random effects meta-analysis of India and Tanzania trials: Low birthweight. 16

Figure S14. Fixed and random effects meta-analysis of India and Tanzania trials: Small-for-gestational age birth. 17

Figure S15. Fixed and random effects meta-analysis of India and Tanzania trials: Infant death <42 days. 18

Table S1. Primary, secondary and safety outcome definitions 19

Table S2. Exploratory sensitivity analyses analyzing the effects of 500 mg as compared to 1500 mg calcium on timing of preeclampsia onset and the effect on preterm birth restricted to spontaneous births. 21

Table S3. India trial: Effect of calcium regimen on preeclampsia and preterm birth adjusting for baseline anemia status. 22

Table S4. Tanzania trial: Effect of calcium regimen on preeclampsia and preterm birth adjusting for baseline gestational age and HIV status. 23

Table S5. Representativeness of Study Participants 24

References 25

List of Authors

India trial team: Pratibha Dwarkanath, M.Sc., Ph.D, Tinku Thomas, M.Sc., Ph.D., Ryan Fernandez, M.B.L., John Michael Raj, M.Sc.,Nirmala Buggi, M.D., Rani Shobha, M.D., Anura V. Kurpad M.D., Ph.D

Tanzania trial team: Alfa Muhihi, M.D., M.P.H, Shabani M. Kinyogoli, B.Sc, Mohamed Bakari, M.Sc., Mary M. Sando, M.D., M.P.H., Honorati M. Masanja, Ph.D, Andrea B. Pembe, M.D., M,Med., Ph.D.

US trial team: Christopher R. Sudfeld, Sc.D, Blair J. Wylie, M.D., M.P.H, Molin Wang, Ph.D., Nandita Perumal, Ph.D. Christopher P. Duggan, M.D., M.P.H, Wafaie W. Fawzi, M.B.B.S., Dr.P.H

Methods

The non-inferiority margins for preclampsia and preterm birth were set by an independent technical advisory group.[1] Both margins were set using meta-analysis estimates of the effect of high-dose calcium supplementation in pregnancy as compared to placebo in the Hofmeyr et. al Cochrane review.[2] The pooled relative risk of preeclampsia for high-dose calcium supplementation as compared to placebo in the review was 0.45, 95% CI 0.31 to 0.65, while the relative risk for preterm birth was RR 0.76, 95% CI 0.60 to 0.97. As a result, the confidence intervals for the effect of high-dose calcium supplementation on preterm birth were particularly wide (consistent with a 3% to 40% reduction in the risk of preterm birth) and the bound of confidence interval was also close to the null.

For preeclampsia, the technical advisory group set a non-inferiority margin of a RR of 1.54 based on the bound of the 95% CI for the relative risk (RR) of high-dose calcium supplementation versus placebo (RR = 1 / 0.65 bound) as recommended in guidance from the US Food and Drug Administration.[3,4] The same method for setting the margin could not be used for preterm birth due to the high uncertainty in the effect of high-dose calcium supplementation as compared to placebo on preterm birth (1 / 0.97 bound = 1.03 RR margin). The technical advisory group therefore set the non-inferiority margin for preterm birth as a RR of 1.16 to preserve 50% of the effect based on the point estimate for preterm birth (1 / 0.76 = 1.32; 0.32 * 50% = 0.16; margin = 1.16). This method is also commonly used to set the margin in non-inferiority trials.[4]

Figure S1. India trial: Flow diagram for pregnant participants

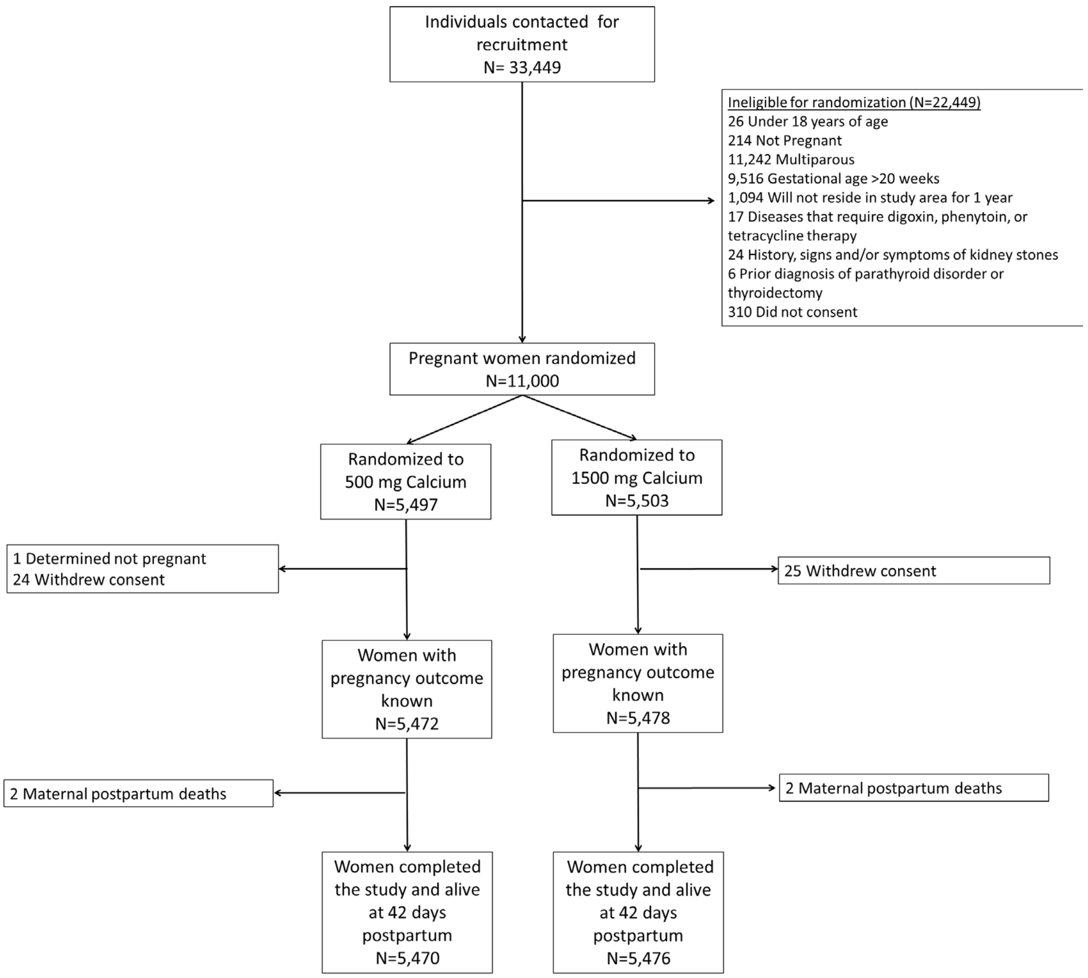

Figure S2. Tanzania trial: Flow diagram for pregnant participants

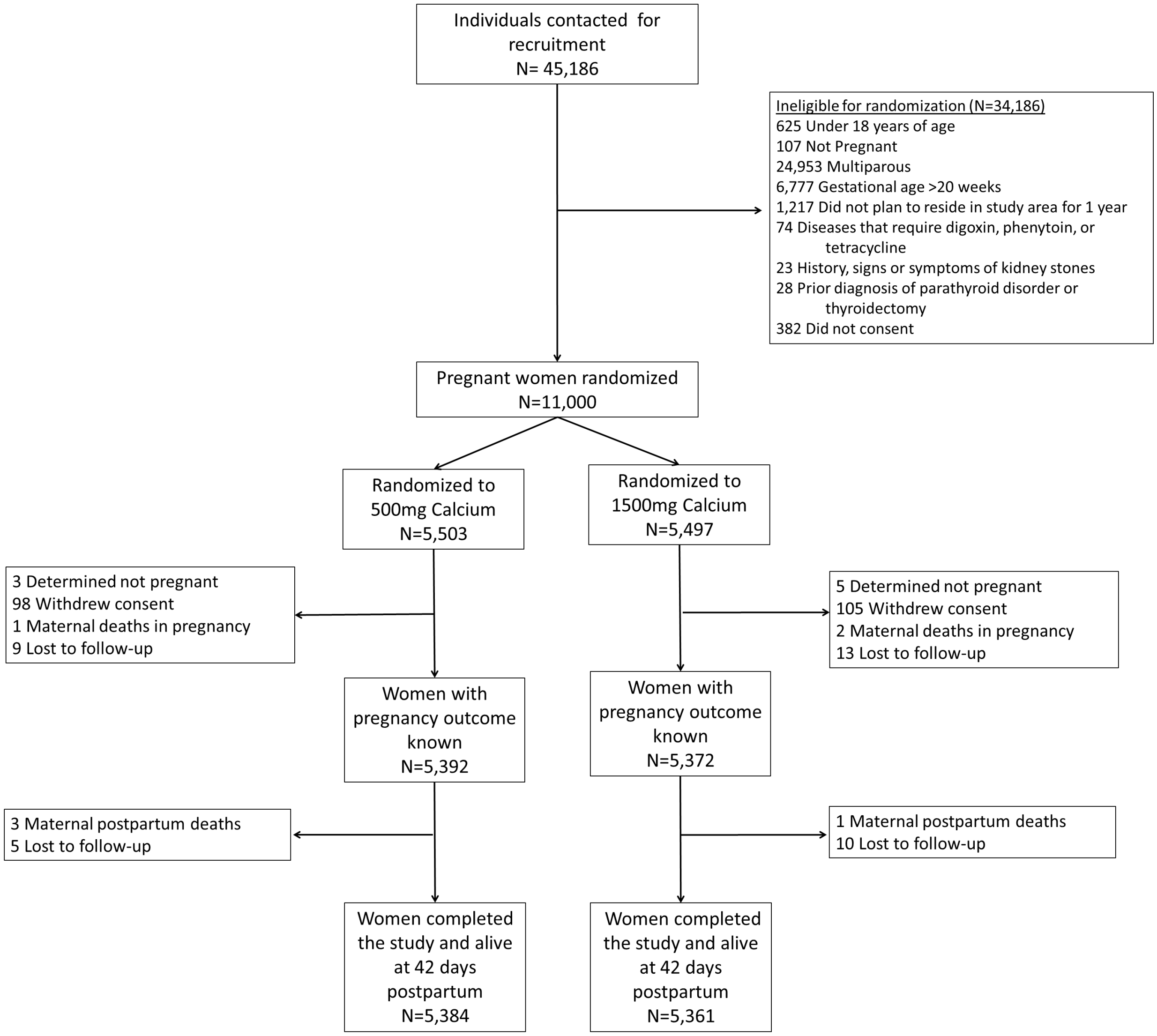

Figure S3. India trial: Kaplan-Meir Curve of the cumulative incidence of preeclampsia by gestational age in weeks stratified by randomized group.

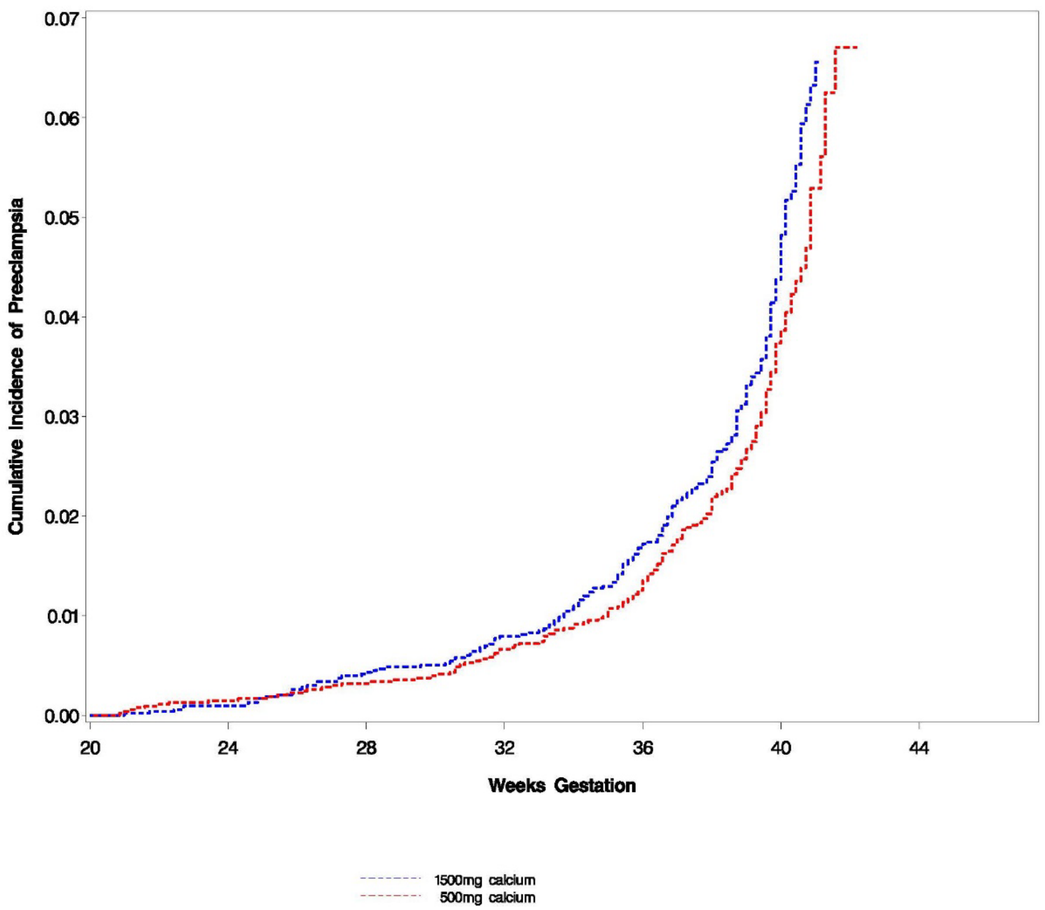

Figure S4. Tanzania trial: Kaplan-Meir Curve of the cumulative incidence of preeclampsia by gestational age in weeks stratified by randomized group.

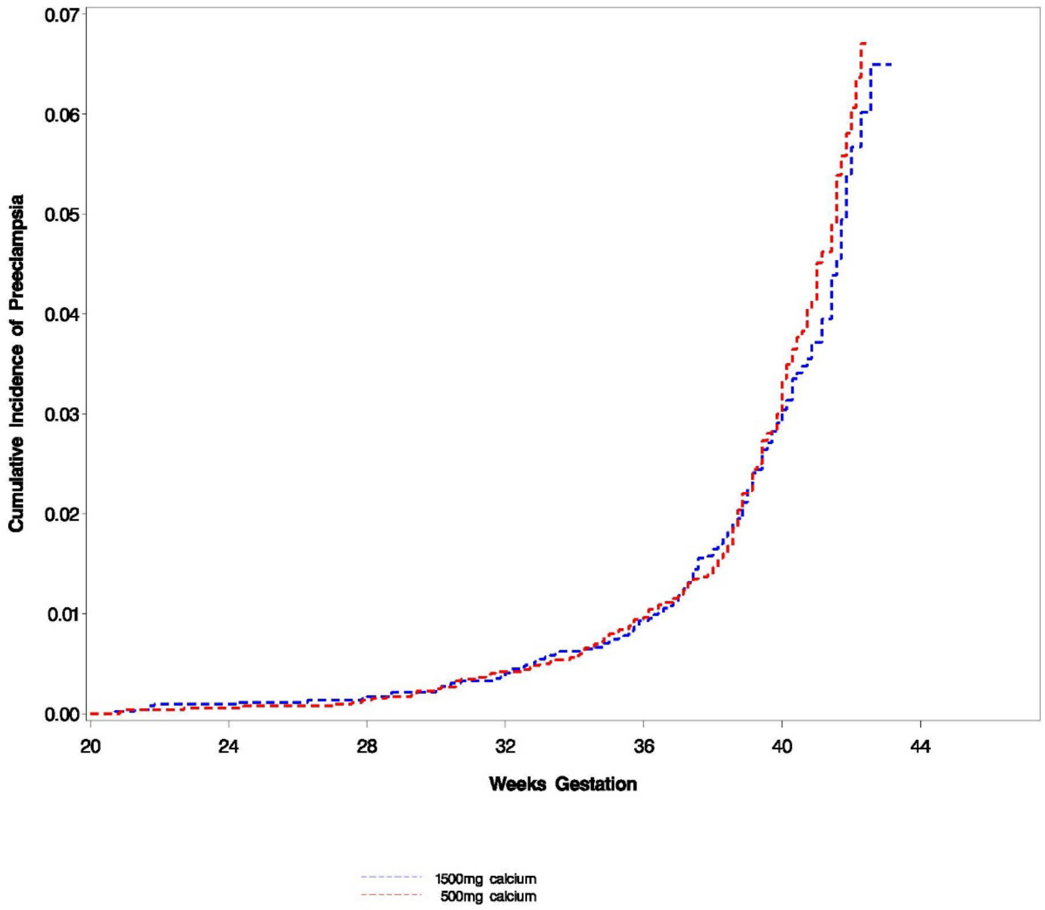

Figure S5. Fixed and random effects meta-analysis of India and Tanzania trials: Preeclampsia. *Meta-analysis estimates are not adjusted for multiplicity and should not be used to infer definitive treatment effects.*

(i) Fixed effects

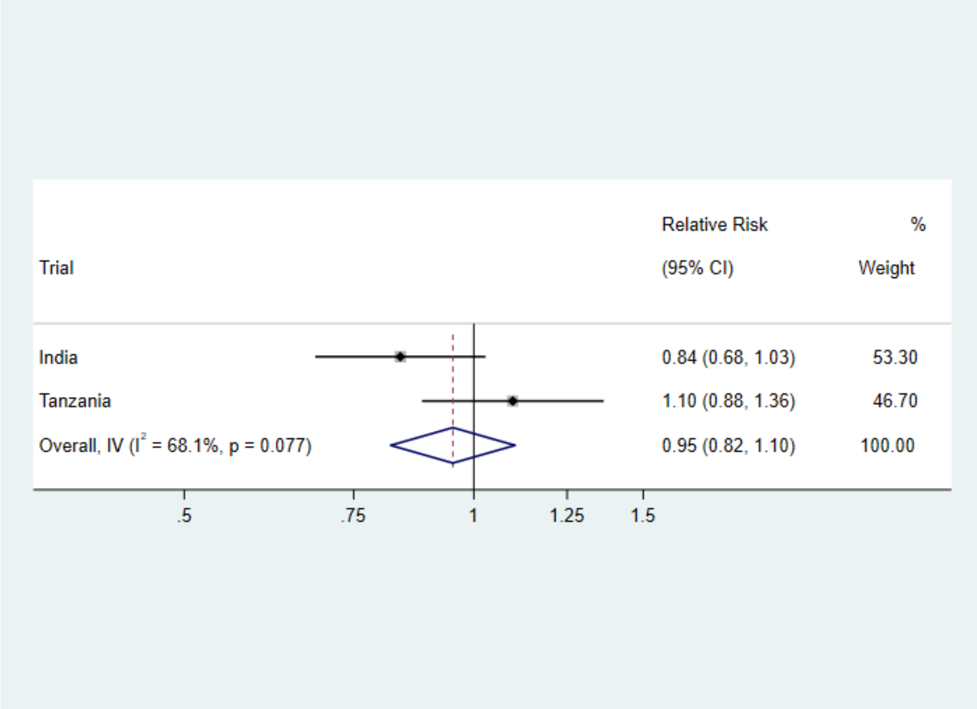

(ii) Random effects

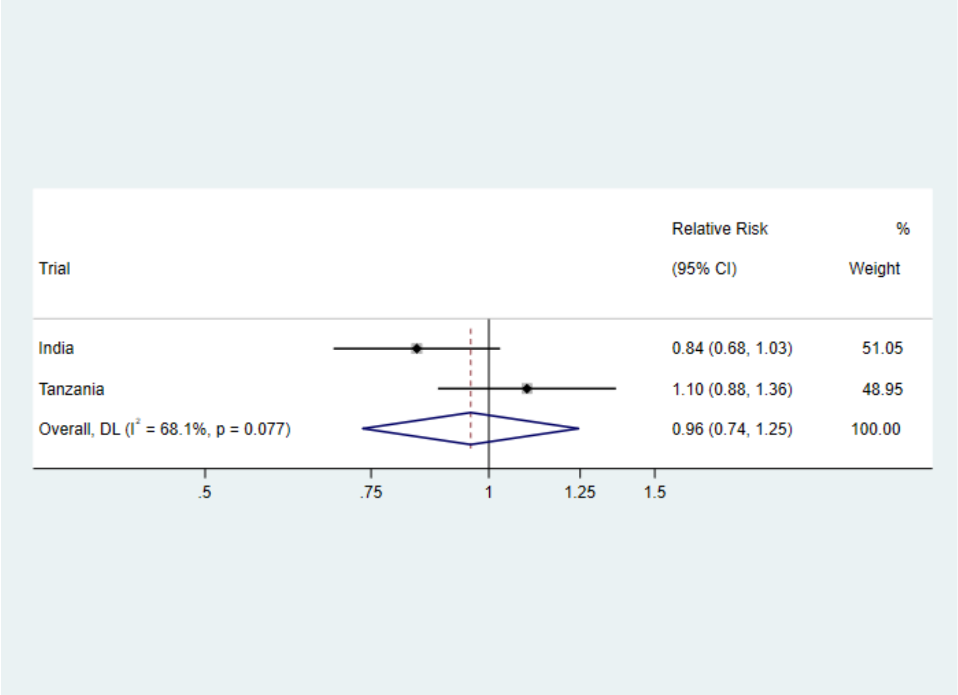

Figure S6. Fixed and random effects meta-analysis of India and Tanzania trials: Preterm birth. *Meta-analysis estimates are not adjusted for multiplicity and should not be used to infer definitive treatment effects.*

(i) Fixed effects

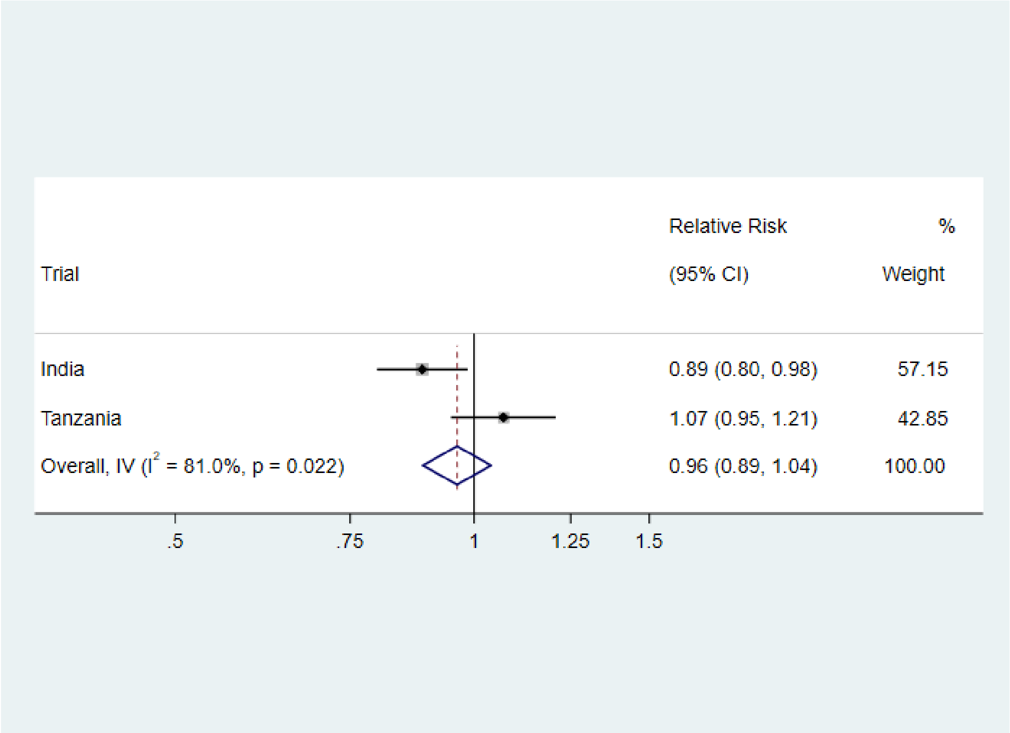

(ii) Random effects

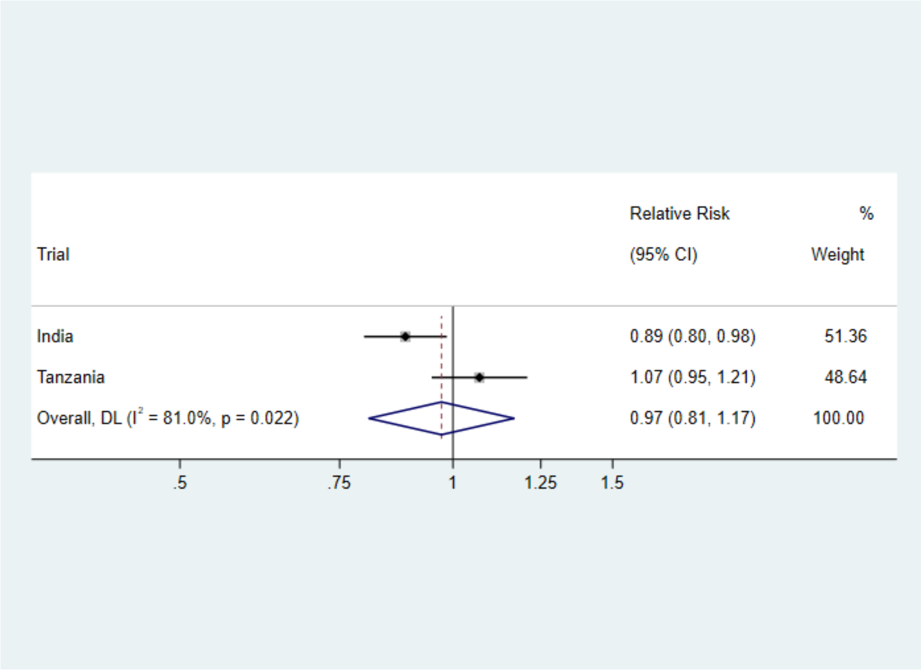

Figure S7. Fixed and random effects meta-analysis of India and Tanzania trials: Gestational hypertension. *Meta-analysis estimates are not adjusted for multiplicity and should not be used to infer definitive treatment effects.*

(i) Fixed effects

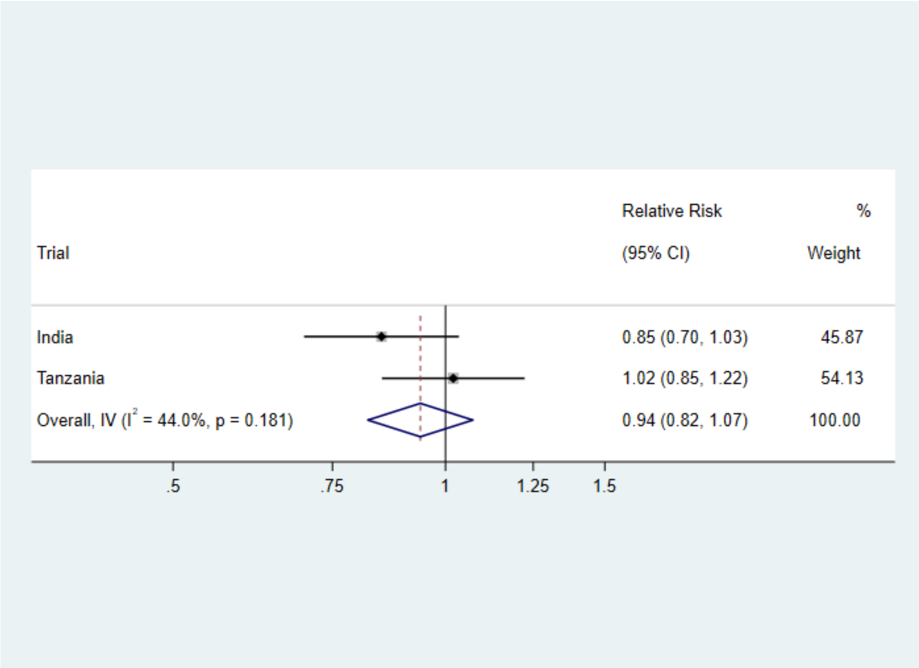

(ii) Random effects

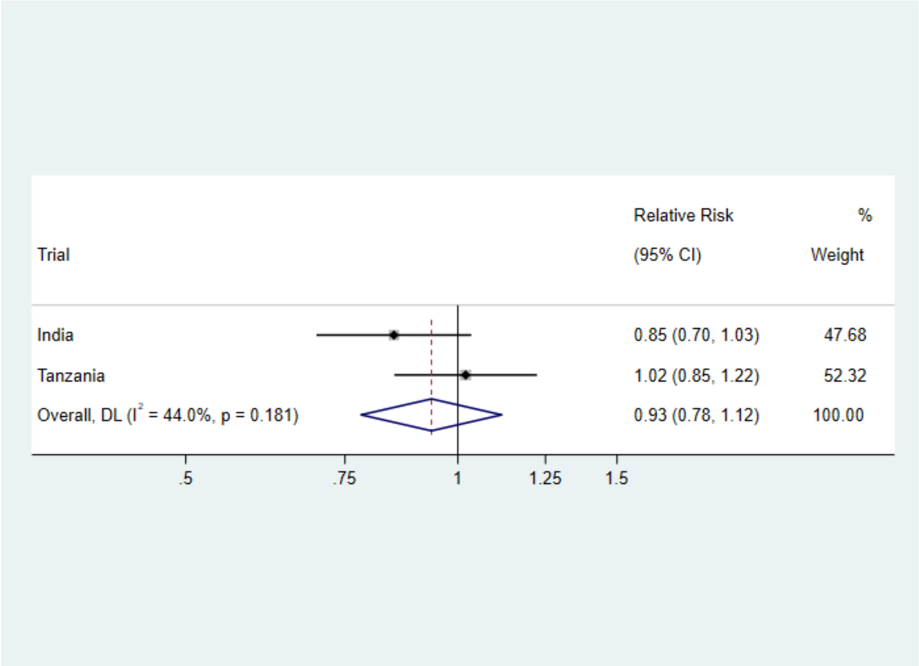

Figure S8. Fixed and random effects meta-analysis of India and Tanzania trials: Preeclampsia with severe features.

Meta-analysis estimates are not adjusted for multiplicity and should not be used to infer definitive treatment effects.

(i) Fixed effect

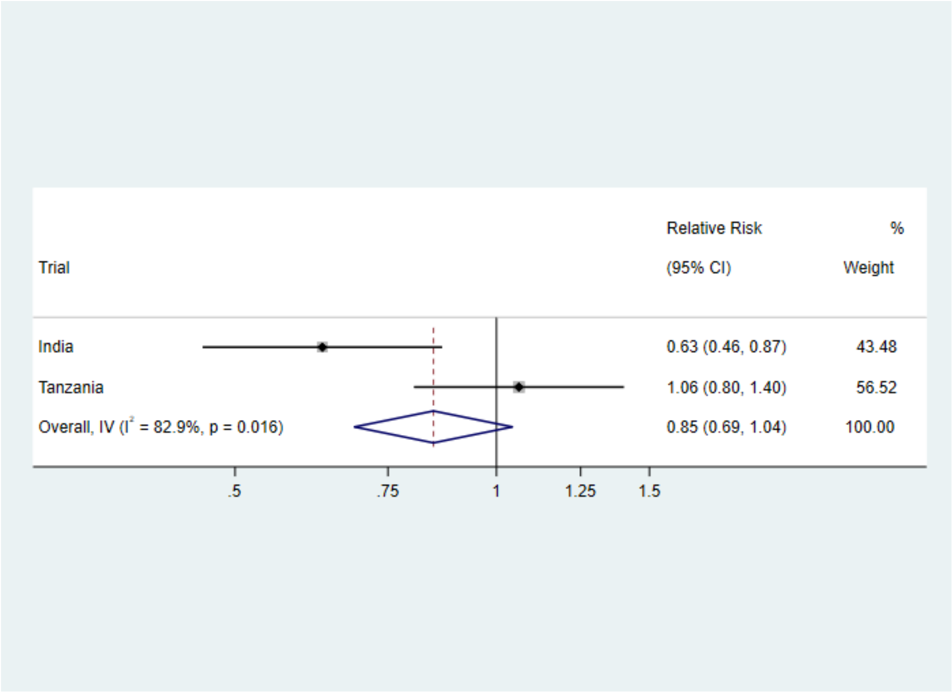

(ii) Random effect

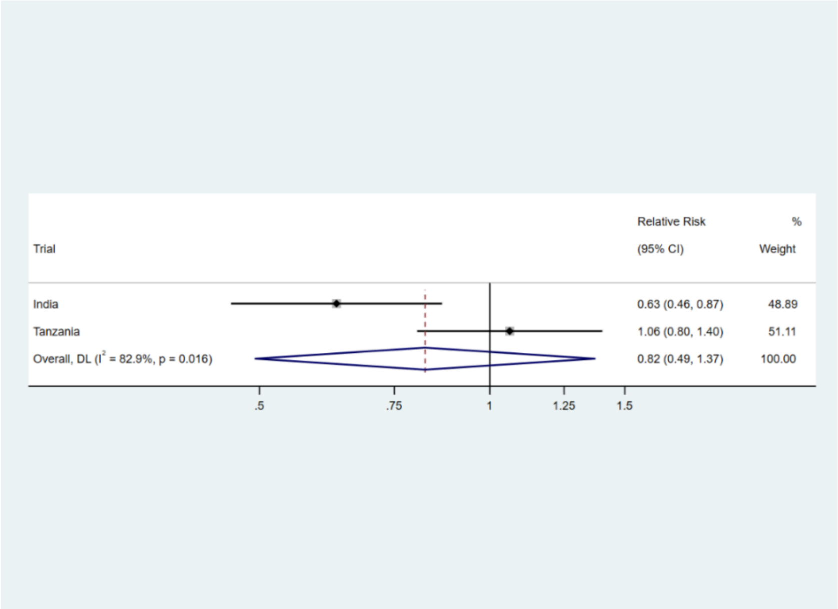

Figure S9. Fixed and random effects meta-analysis of India and Tanzania trials: Maternal hospitalization. *Meta-analysis estimates are not adjusted for multiplicity and should not be used to infer definitive treatment effects.*

(i) Fixed effect

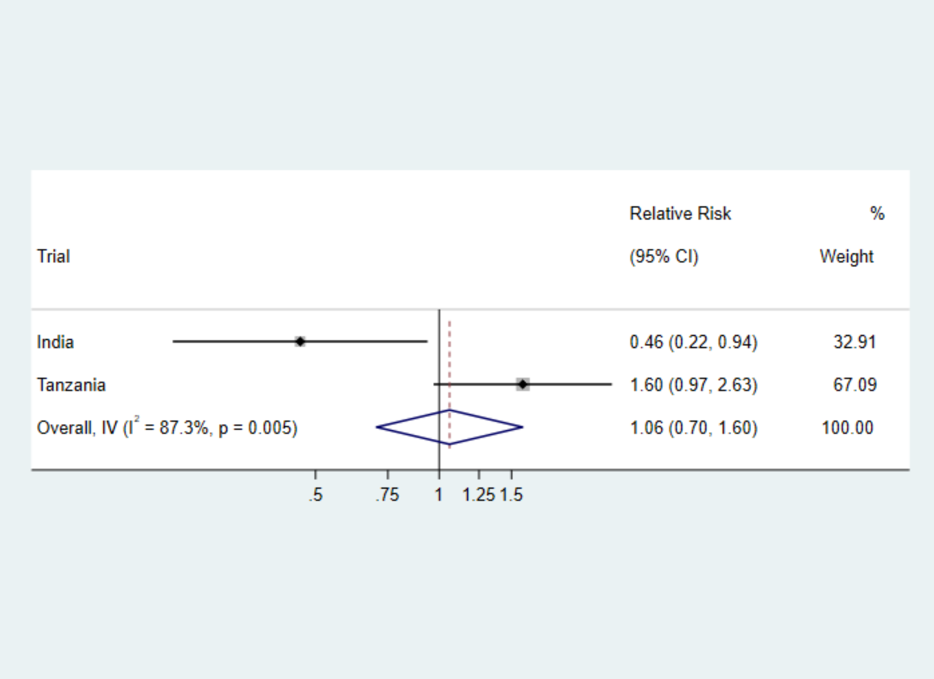

(ii) Random effect

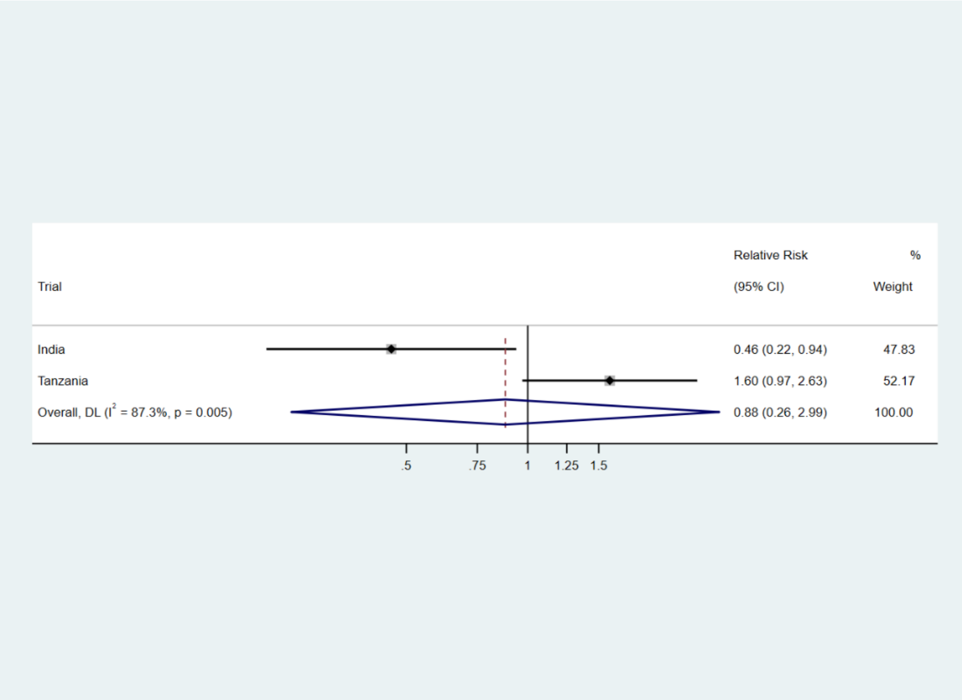

Figure S10. Fixed and random effects meta-analysis of India and Tanzania trials: Maternal death. *Meta-analysis estimates are not adjusted for multiplicity and should not be used to infer definitive treatment effects.*

(i) Fixed effect

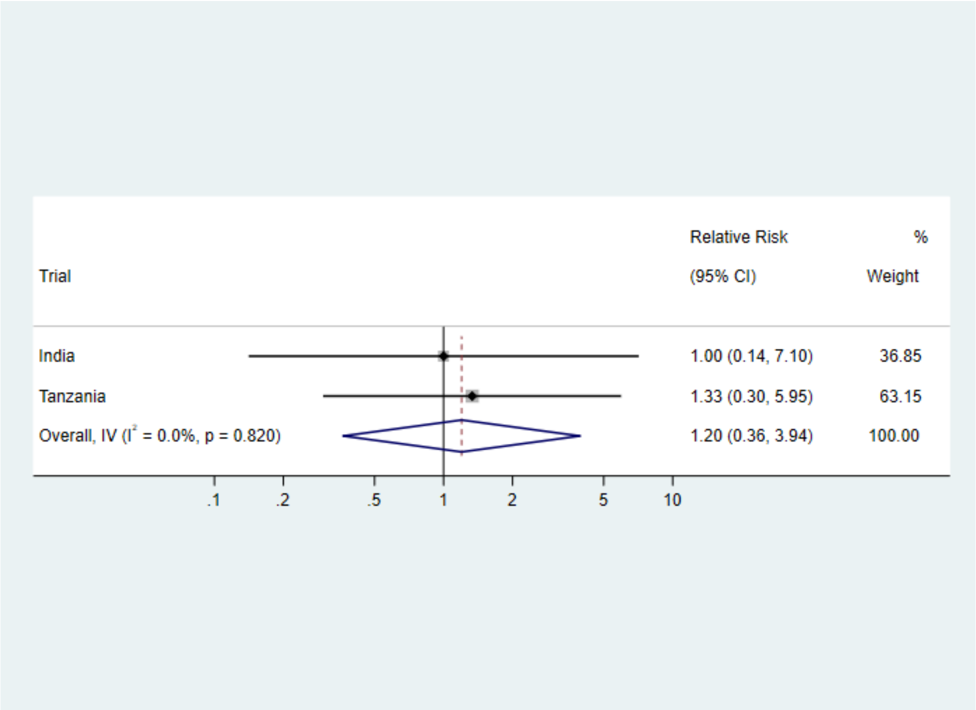

(ii) Random effect

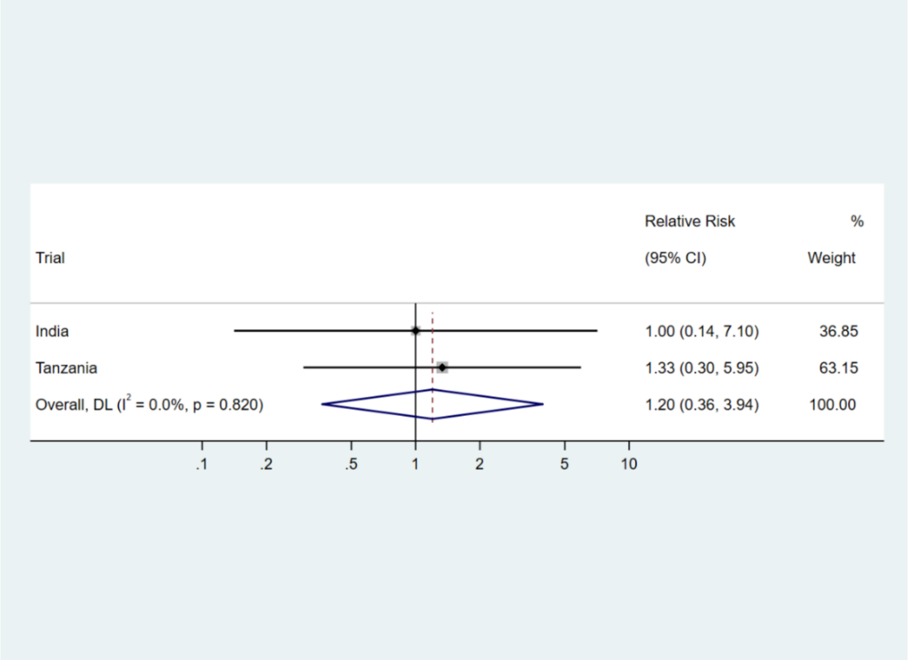

Figure S11. Fixed and random effects meta-analysis of India and Tanzania trials: Fetal death.
Meta-analysis estimates are not adjusted for multiplicity and should not be used to infer definitive treatment effects.

(i) Fixed effect

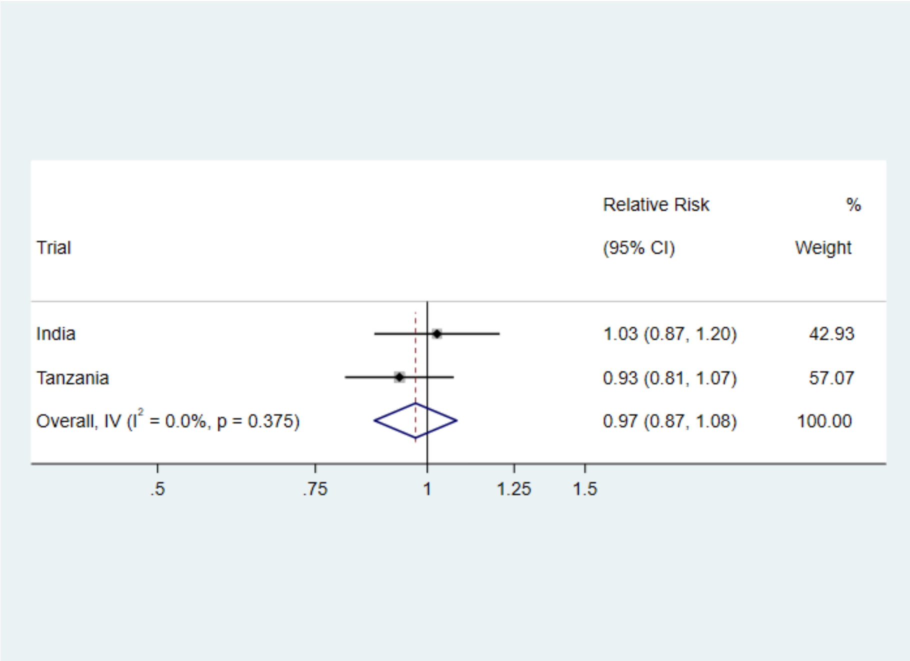

(ii) Random effect

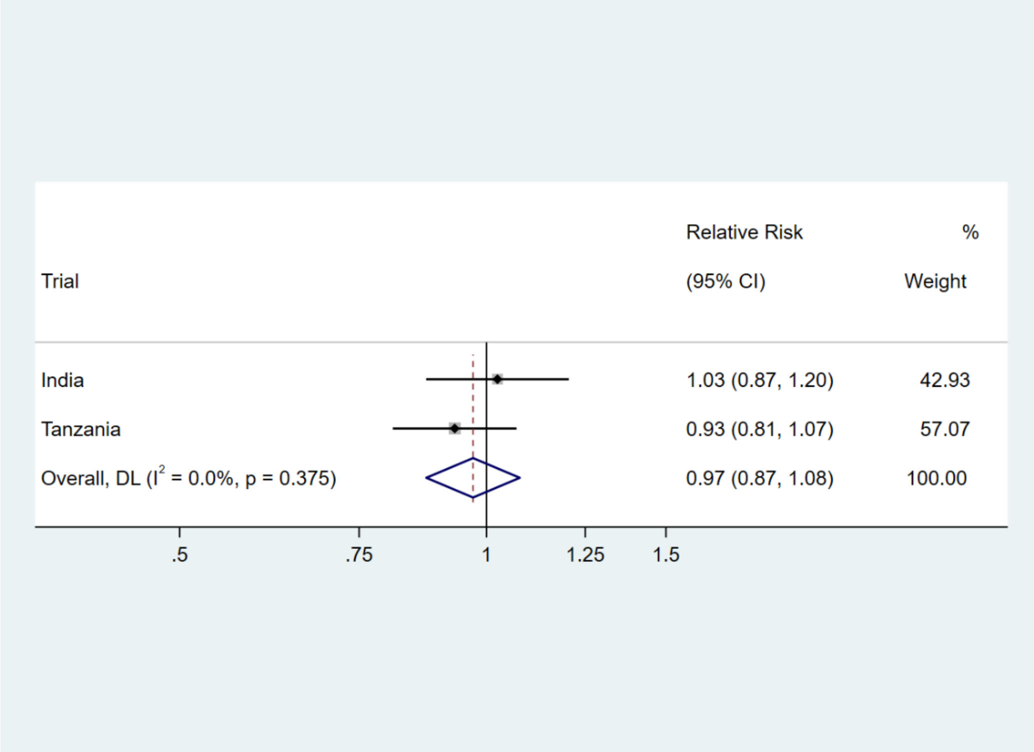

Figure S12. Fixed and random effects meta-analysis of India and Tanzania trials: Stillbirth. *Meta-analysis estimates are not adjusted for multiplicity and should not be used to infer definitive treatment effects.*

(i) Fixed effect

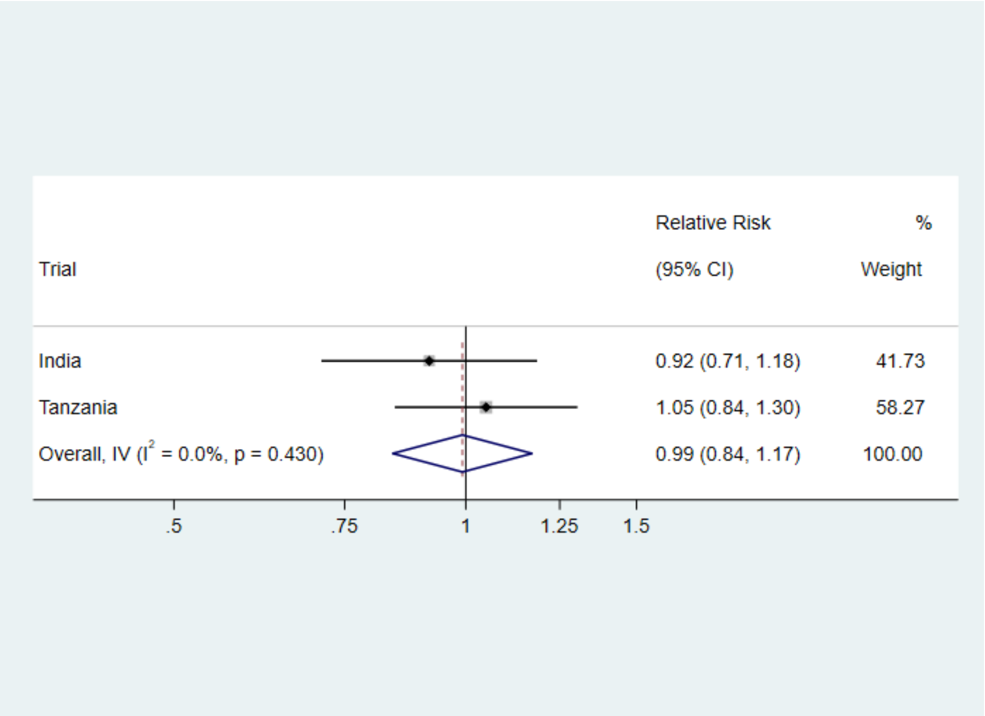

(ii) Random effect

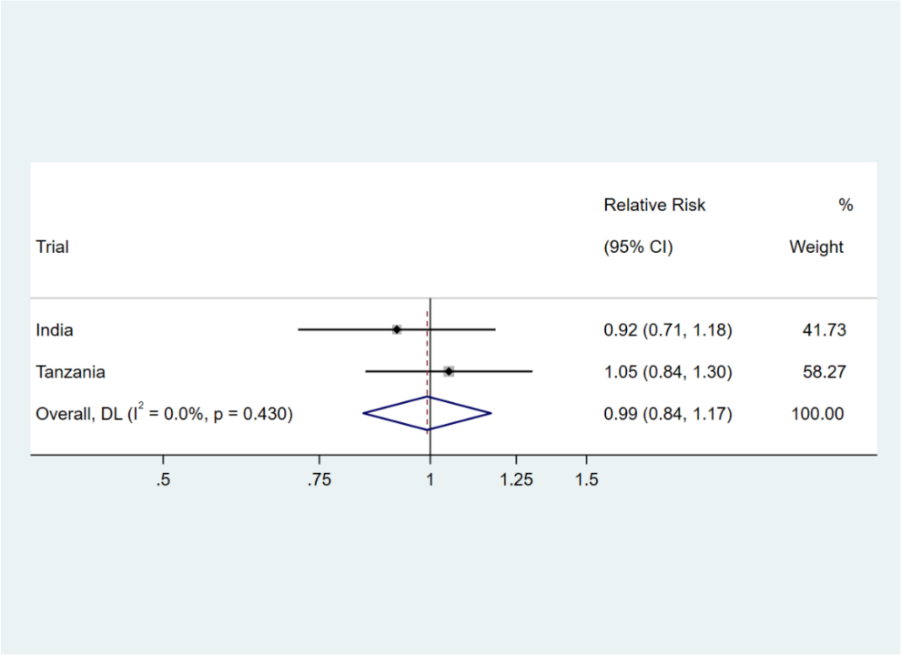

Figure S13. Fixed and random effects meta-analysis of India and Tanzania trials: Low birthweight. *Meta-analysis estimates are not adjusted for multiplicity and should not be used to infer definitive treatment effects.*

(i) Fixed effect

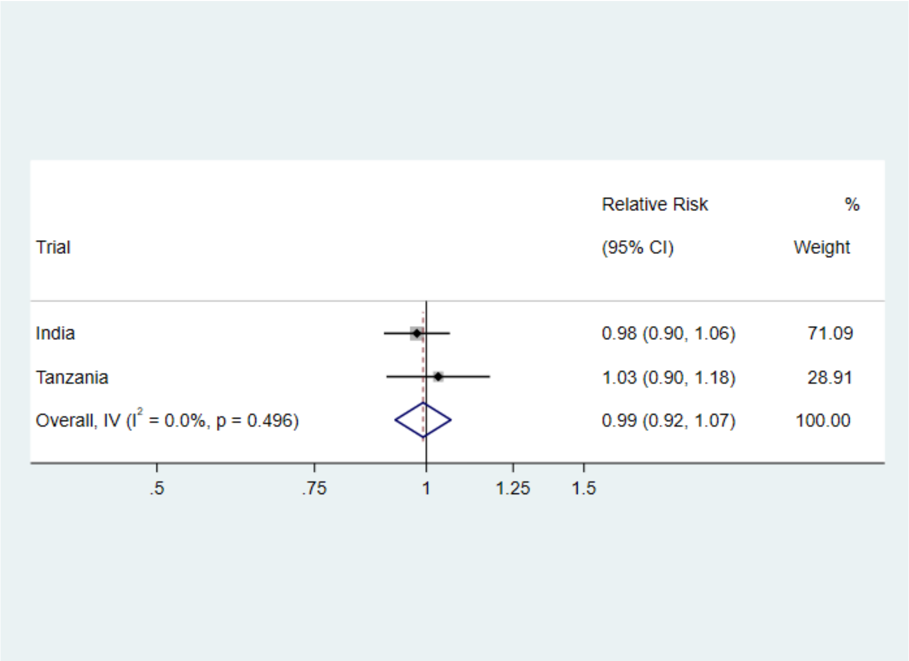

(ii) Random effect

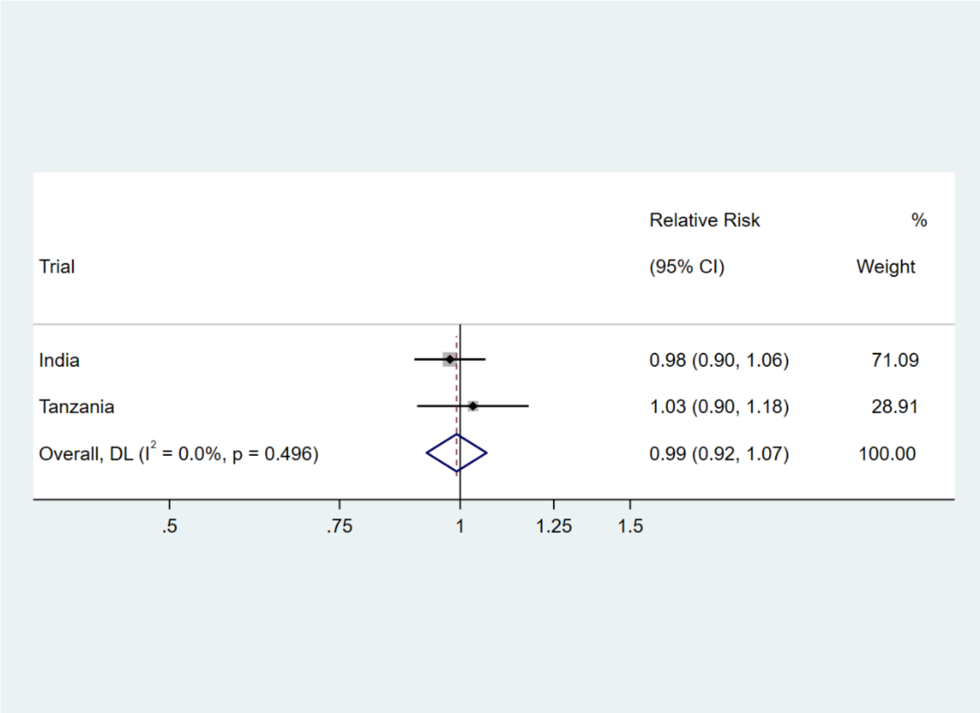

Figure S14. Fixed and random effects meta-analysis of India and Tanzania trials: Small-for-gestational age birth.

Meta-analysis estimates are not adjusted for multiplicity and should not be used to infer definitive treatment effects.

(i) Fixed effect

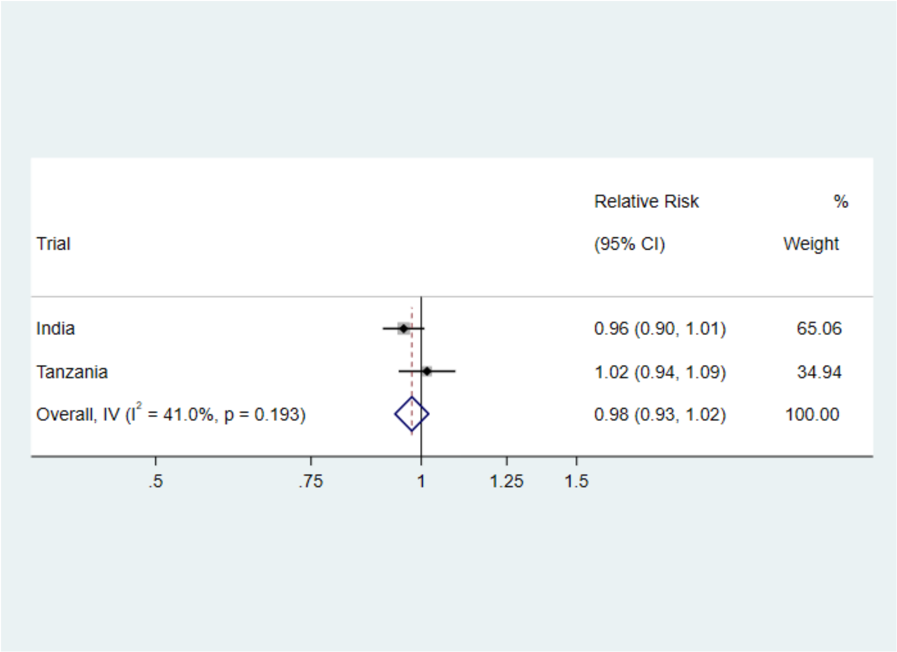

(ii) Random effect

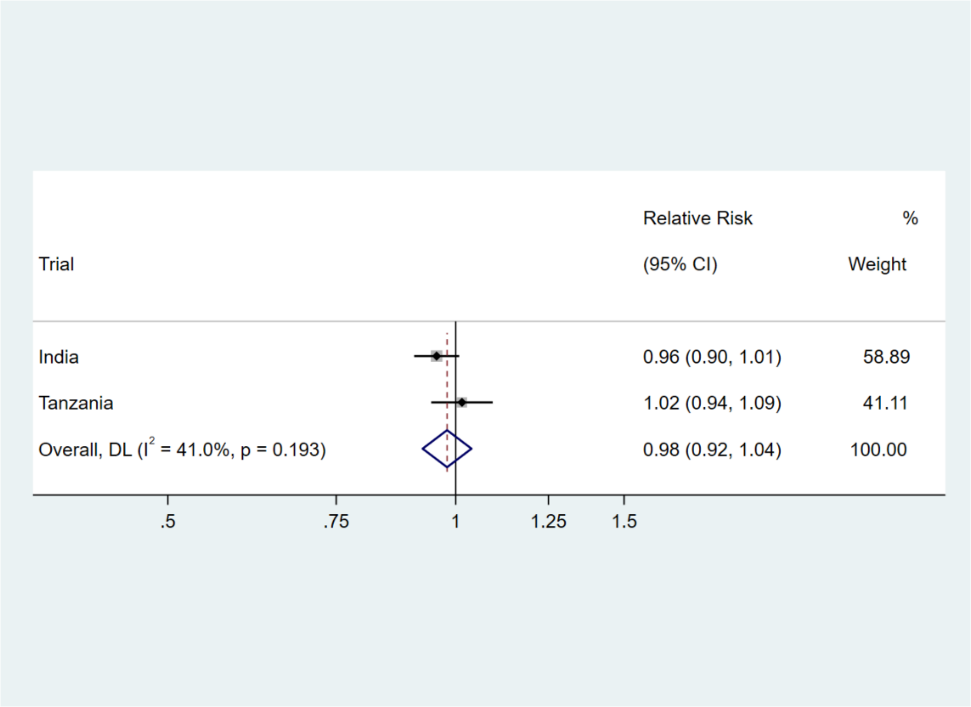

Figure S15. Fixed and random effects meta-analysis of India and Tanzania trials: Infant death <42 days. *Meta-analysis estimates are not adjusted for multiplicity and should not be used to infer definitive treatment effects.*

(i) Fixed effect

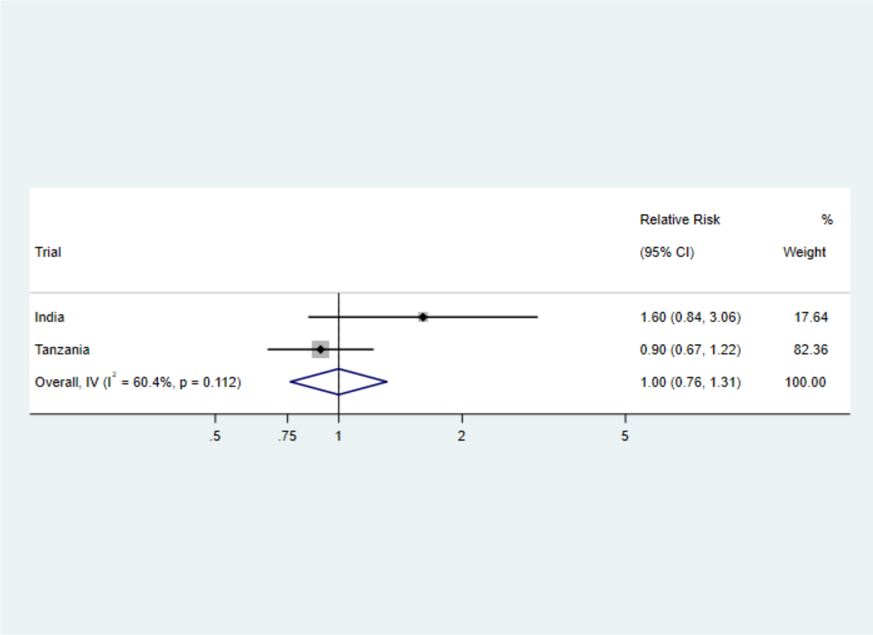

(i) Random effect

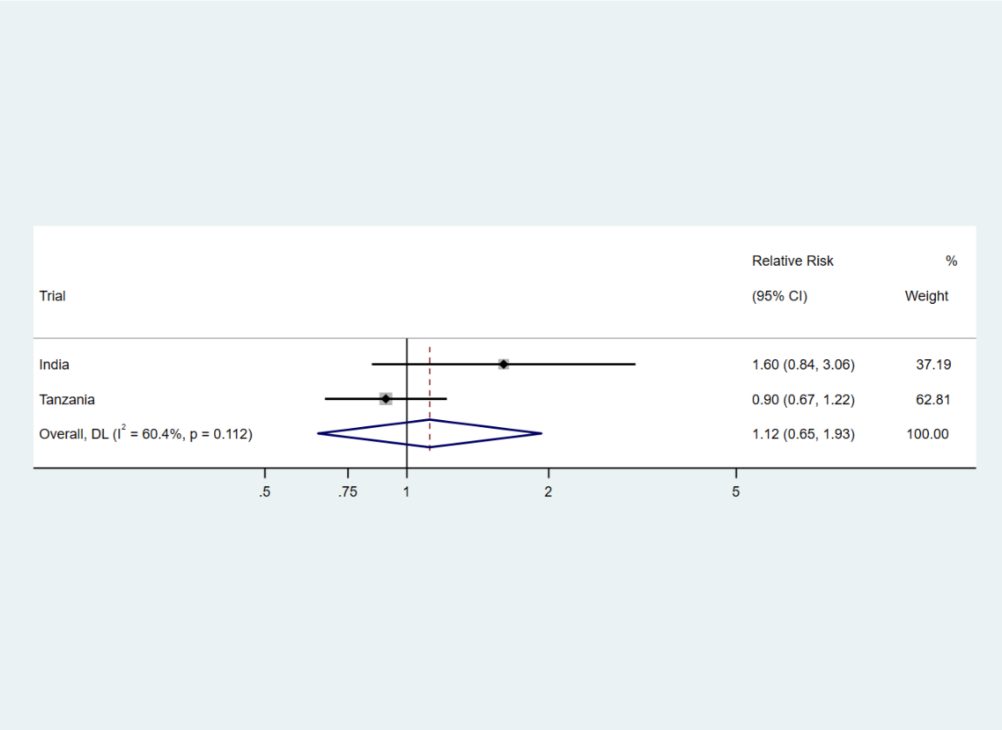

Table S1. Primary, secondary and safety outcome definitions

**Primary Outcomes**	**Definition**	**Analytic Population**
Preeclampsia	Gestational hypertension and gestational proteinuria among participants without chronic hypertension; or, gestational proteinuria among participants with chronic hypertension (superimposed preeclampsia); or, clinical diagnosis of preeclampsia by managing clinical team; or, development of severe features of preeclampsia with or without proteinuria. The timeperiod was from randomization to delivery.	Randomized pregnant women
Preterm birth	Live birth <37 weeks gestation	Livebirths, including multiple gestation<br><br>(fetal deaths excluded)
**Secondary and Safety Outcomes**	**Definition**	**Analytic Population**
Gestational hypertension	A systolic blood pressure ≥140 mm Hg on two occasions at least 1 hour apart; or, diastolic blood pressure ≥90 mm Hg on two occasions at least 1 hour apart; or, severe gestational hypertension in pregnancy among participants without chronic hypertension, defined in the antenatal period as systolic blood pressure ≥160 mmHg or a diastolic blood pressure ≥110 mmHg on two occasions at least 1 minute apart after 20 weeks gestation and defined at the time of labor/delivery as requiring only one systolic blood pressure ≥160 mmHg or a diastolic blood pressure ≥110 mmHg.<br><br>Note: Chronic hypertension defined by a clinical diagnosis of chronic hypertension (with or without need for medication) or systolic blood pressure ≥140 mm Hg on two occasions at least 1 hour apart or diastolic blood pressure ≥90 mm Hg on two occasions at least 1 hour apart before 20 weeks' gestation.	Randomized pregnant women
Severe features of preeclampsia	The following will be defined as severe features of preeclampsia:<br>• Severe gestational hypertension (with or without proteinuria), or<br>• Eclampsia, or<br>• Evidence of end organ dysfunction by laboratory criteria, or<br>• Clinical diagnosis of HELLP syndrome (hemolysis, elevated liver function, low platelets), or<br>• development of pulmonary edema, or<br>• new onset CNS or visual symptoms	Randomized pregnant women
Pregnancy-related death	Death of a woman while pregnant or within 42 days of termination of pregnancy, irrespective of the cause of death	Randomized pregnant women
Third trimester severe anemia [safety outcome]	Hemoglobin concentration < 7.0 g/dL	Randomized pregnant women

Maternal hospitalization [safety outcome]	Maternal reported overnight stay in hospital during pregnancy or up to 42 days postpartum. Excludes delivery hospitalization.	Randomized pregnant women
Fetal death	A product of human conception, irrespective of the duration of the pregnancy, which, after expulsion or extraction, does *not* breath or show any other evidence of life such as beating of the heart, pulsation of the umbilical cord, or definite movement of voluntary muscles, whether or not the umbilical cord has been cut or the placenta is attached	Fetuses, including multiple gestation
Stillbirth	Fetal death ≥ 28 weeks gestation	Stillbirths and livebirths, including multiple gestation<br><br>(Excludes fetal deaths before 28 weeks and medically induced abortions)
Low birthweight	Livebirth weighing <2500 g	Livebirths, including multiple gestation<br><br>(fetal deaths excluded)
Small-for-gestational age birth	Live birth with size-for-gestational age <10th percentile on the INTERGROWTH-21st standard	Livebirths, including multiple gestation<br><br>(fetal deaths excluded)
Infant death < 42 days	Death of a live birth during the first 42 completed days of life (6 weeks postpartum)	Livebirths, including multiple gestation<br><br>(fetal deaths excluded)

Table S2. Exploratory sensitivity analyses analyzing the effects of 500 mg as compared to 1500 mg calcium on timing of preeclampsia onset and the effect on preterm birth restricted to spontaneous births. *Estimates are not adjusted for multiplicity and should not be used to infer definitive treatment effects*

	**India Trial**			**Tanzania Trial**		
	**500mg Calcium**	**1500mg Calcium**	**Relative risk (Two-sided 95% CI)**	**500mg Calcium**	**1500mg Calcium**	**Relative risk (Two-sided 95% CI)**
Early-onset preeclampsia <34 weeks gestation	46 / 5,497 (0.8%)	56 / 5,503 (1.0%)	0.82 (0.56, 1.21)	29 / 5,503 (0.5%)	32 / 5,497 (0.6%)	0.90 (0.55, 1.49)
Preeclampsia onset <37 weeks gestation	87 / 5,497 (1.6%)	107 / 5,503 (1.9%)	0.81 (0.61, 1.08)	58 / 5,503 (1.1%)	56 / 5,497 (1.0%)	1.03 (0.72, 1.49)
Preterm birth <37.0 weeks gestation among spontaneous livebirths*	334 / 2,884 (11.6%)	371 / 2,830 (13.1%)	0.89 (0.77, 1.02)	480 / 4,729 (10.2%)	444 / 4,724 (9.4%)	1.07 (0.94, 1.22)

*Excludes data from induced live births (3,220 in India trial and 554 in Tanzania trial) and live births that spontaneous/induced status was unknown (1,454 in India trial and 183 in Tanzania trial)

Table S3. India trial: Effect of calcium regimen on preeclampsia and preterm birth adjusting for baseline anemia status.

Estimates are not adjusted for multiplicity and should not be used to infer definitive treatment effects

	**Relative risk 500mg versus 1500 mg (Two-sided 95% CI)**
Preeclampsia	0.84 (0.68, 1.03)
Preterm birth	0.89 (0.80, 0.99)

Table S4. Tanzania trial: Effect of calcium regimen on preeclampsia and preterm birth adjusting for baseline gestational age and HIV status.

Estimates are not adjusted for multiplicity and should not be used to infer definitive treatment effects

	**Relative risk 500mg versus 1500 mg (Two-sided 95% CI)**
Preeclampsia	1.10 (0.88, 1.36)
Preterm birth	1.08 (0.95, 1.22)

Table S5. Representativeness of Study Participants

Category	
Disease, problem or conduction under investigation	1) Preeclampsia<br>2) Preterm birth
Special considerations	
Sex and Gender	Preeclampsia and preterm birth affect pregnant populations
Age	A maternal age of 35 years or greater is a risk factor for pre-eclampsia and preterm birth. Adolescent pregnancies are also at increased risk for preeclampsia and preterm birth.
Race or ethnic group	Global evidence of the contributions of race and ethnicity to the risk of pre-eclampsia and preterm birth is limited. There is evidence of differential risk of preeclampsia and preterm birth in some countries by race and ethnic groups. As example in the United States, non-Hispanic Black pregnant persons have a greater risk of preeclampsia and preterm birth as compared to non-Hispanic White pregnant persons. While rates of preeclampsia and preterm birth are known differ between populations globally, the causes and potential contributions of genetics, disproportionate burden of risk factors for preeclampsia and preterm birth (i.e., chronic hypertension, high body mass index, infections, etc.), and other factors to explain the differences in risk are not fully characterized.
Other considerations	Parity is associated with the risk of pre-eclampsia and preterm birth. Nulliparous pregnant persons are at increased risk for pre-eclampsia and preterm birth.
Overall representativeness of the trials	The participants in the present calcium supplementation trials conducted in India and Tanzania enrolled adult (18+ years) nulliparous pregnant participants. Given nulliparity was an enrollment criterion, pregnant participants tended to be younger in age as compared to the general population of pregnancies in the study settings; the majority of pregnant participants were 18-24 years of age in both India (~62%) and Tanzania (~70%) trials. Likely due to young age, the prevalence of chronic hypertension was also lower than the general population of pregnancies in both settings. Therefore, care should be taken when considering the generalizability of our findings to pregnant populations that differ in parity, age, and chronic hypertension risk. We did not ask participants to report their gender identity or their race or ethnic identity.

References

1. Dwarkanath P, Muhihi A, Sudfeld CR, et al. Non-inferiority of low-dose compared to standard high-dose calcium supplementation in pregnancy: study protocol for two randomized, parallel group, non-inferiority trials in India and Tanzania. Trials 2021;22:838.

2. Hofmeyr GJ, Lawrie TA, Atallah AN, Torloni MR. Calcium supplementation during pregnancy for preventing hypertensive disorders and related problems. Cochrane Database Syst Rev 2018;10:CD001059.

3. Center for Drug Evaluation and Research, Center for Biologics Evaluation and Research. Non-inferiority clinical trials to establish effectiveness — guidance for industry. Silver Spring, MD: Food and Drug Administration, November 2016.

4. Mauri L, D'Agostino RB, Sr. Challenges in the Design and Interpretation of Noninferiority Trials. N Engl J Med 2017;377:1357-67.
